# The KLDpT activation loop motif is critical for MARK kinase activity

**DOI:** 10.1371/journal.pone.0225727

**Published:** 2019-12-03

**Authors:** Tim Sonntag, James J. Moresco, John R. Yates, Marc Montminy

**Affiliations:** 1 Clayton Foundation Laboratories for Peptide Biology, The Salk Institute for Biological Studies, La Jolla, California, United States of America; 2 Department of Molecular Medicine, The Scripps Research Institute, La Jolla, California, United States of America; McGill University, CANADA

## Abstract

MAP/microtubule-affinity regulating kinases (MARK1-4) are members of the AMPK family of Ser/Thr-specific kinases, which phosphorylate substrates at consensus LXRXXSXXXL motifs. Within microtubule-associated proteins, MARKs also mediate phosphorylation of variant KXGS or ζXKXGSXXNΨ motifs, interfering with the ability of tau and MAP2/4 to bind to microtubules. Here we show that, although MARKs and the closely related salt-inducible kinases (SIKs) phosphorylate substrates with consensus AMPK motifs comparably, MARKs are more potent in recognizing variant ζXKXGSXXNΨ motifs on cellular tau. In studies to identify regions of MARKs that confer catalytic activity towards variant sites, we found that the C-terminal kinase associated-1 (KA1) domain in MARK1-3 mediates binding to microtubule-associated proteins CLASP1/2; but this interaction is dispensable for ζXKXGSXXNΨ phosphorylation. Mutational analysis of MARK2 revealed that the N-terminal kinase domain of MARK2 is sufficient for phosphorylation of both consensus and variant ζXKXGSXXNΨ sites. Within this domain, the KLDpT activation loop motif promotes MARK2 activity both intracellularly and *in vitro*, but has no effect on SIK2 activity. As KLDpT is conserved in all vertebrates MARKs, we conclude that this sequence is crucial for MARK-dependent regulation of cellular polarity.

## Introduction

5' AMP-activated protein kinase (AMPK) family members share a high degree of sequence similarity within the N-terminal kinase domains [1], but their regulatory C-termini are far more divergent. Phosphorylation of a conserved threonine residue within the activation loop is necessary for the activation of all AMPK family members, whereupon they regulate various cellular processes including metabolism, growth, and polarity [2]. The tumor suppressor liver kinase B1 (LKB1 or STK11) activates all 13 AMPK family members: AMPKα1/α2 (PRKAA1/2), BRSK1/2, NUAK1/2, SIK1-3, MARK1-4 [3], as well as the related SNRK [4]. With the exception of AMPKs and BRSKs, the interaction with LKB1 is mediated by the ubiquitin-associated (UBA) domain, which is located directly adjacent to the kinase domain [5]. Some AMPK subfamilies can also be activated by other signal-dependent kinases. For example, MARKs are substrates of genotoxic stimuli-sensing serine/threonine-protein kinase TAO1 (TAOK1) [6, 7] and AMPK is phosphorylated by calcium-sensing CAMKK2 [8].

Consistent with their sequence homology within the kinase domain [1], the Ser/Thr-specific kinases of the AMPK family recognize substrates carrying a common LXRXXSXXXL phosphorylation motif [2]. Indeed, the most closely related subfamilies of salt-inducible kinases (SIKs; SIK1-3) and MAP/microtubule-affinity regulating kinases (MARKs; MARK1-4) can both phosphorylate cAMP-regulated transcriptional coactivators (CRTCs; CRTC1-3) as well as class IIA histone deacetylases (HDACs; HDAC4,5,7,9) at conserved AMPK substrate motifs, promoting phosphorylation-dependent 14-3-3 protein interactions and cytoplasmic sequestration [9–13]. Consistent with their divergent regulatory C-terminal regions, SIK, but not MARK, activity is inhibited by increases in cyclic AMP (cAMP) signaling, where protein kinase A (PKA)-mediated phosphorylation induces 14-3-3 protein binding and inhibition of SIK catalytic activity [11]. By contrast, MARKs interact with cell membranes via their C-terminal kinase associated-1 (KA1) domain [14]; this interaction is disrupted by atypical protein kinase C (aPKC)-mediated phosphorylation and binding of 14-3-3 proteins [15, 16]. In line with these effects, SIKs and MARKs perform distinct biological roles: SIKs regulate glucose and lipid metabolism [17], whereas MARKs modulate cellular polarity [18].

The effects of MARKs on polarity and growth have been linked to their ability to regulate the microtubule association of tau (MAPT) and Microtubule Associated Proteins 2 and 4 (MAP2/4) [19]. When bound to microtubules, these neuronally enriched proteins promote microtubule assembly and stabilization [20], which can be blocked by phosphorylation at KXGS motifs within microtubule-binding repeat (R) domains [19]. MARK2 phosphorylates tau (the Tau-441 or 2N4R isoform) at four KXGS motifs, with S262, S324, and S356 representing high affinity sites *in vitro* [21]. In neurodegenerative diseases known as tauopathies, such as Alzheimer disease (AD), tau forms intra- and extracellular aggregates that are classified as paired helical filaments (PHFs) and neurofibrillary tangles (NFTs) [20]. Although tau contains 45 experimentally observed phosphorylation sites [22], many of which are hyperphosphorylated in AD brains [23], phosphorylation at KXGS sites actually prevents tau aggregation and blocks assembly into PHFs [24]. In addition to its application as an AD biomarker [25], tau is apparently required for amyloid beta (Aβ)-dependent neurotoxicity [26] and serves as a target for ongoing AD drug discovery [27].

Other AMPK family members can also phosphorylate tau at KXGS motifs, including AMPK, BRSKs, NUAKs, and SIKs [28–32], prompting us to investigate whether closely related MARK and SIK subfamilies phosphorylate KXGS motifs to the same extent. Although they have comparable effects on AMPK substrate motif phosphorylation in CRTCs and class IIA HDACs, MARKs are more potent than SIKs with respect to the phosphorylation of cellular KXGS motifs. Mutational analysis uncovered a critical MARK-specific KLDpT activation loop motif, which is required for activity both intracellularly and *in vitro*. These results point to vertebrate MARKs as major regulators of tau and MAPs binding to microtubules.

## Materials and methods

The methods were previously described and are reprinted here, mostly verbatim, for reference [10, 11].

### Small molecules

Small molecules were solubilized in DMSO (ACS, Sigma-Aldrich) at the indicated concentrations and stored until usage at -80°C (long term storage) or -20°C (working dilution): 20 mM Forskolin (Sigma-Aldrich), 10 mM Doxorubicin (Cayman Chemical; light protected), and 2 mM Carfilzomib (PR-171; Selleck Chemicals).

### Antibodies

The antibodies used in Western blot analysis were purchased from Abcam PLC (CLASP1 # ab108620, TAU-5 #ab80579, Tau & P-Tau S262 #ab64193, P-Tau S262 & T263 #ab92627, P-Tau S324 #ab109401, P-Tau S610 #ab192030), Cell Signaling Technology (14-3-3 [pan] #8312, P-CREB S133 #9198, P-CRTC2 S171 #2892, HDAC4 #7628, P-HDAC4(5/7) S246 #3443, P-HDAC4(5/7) S632 #3424, MARK2 #9118, P-MARK AL [AL = activation loop] #4836, SIK2 #6919), Covance (GFP #MMS-118P), EMD Millipore (α-tubulin clone DM1A), Invitrogen (P-Tau S262 #44-750G), Qiagen (Penta-His #34660), Roche (Anti-HA-Peroxidase clone 3F10), and Sigma-Aldrich (FLAG M2 #F1804).

The antisera for P-CRTC3 S273 (PBL #7378) [10], CRTC2 (PBL #6896) [33], P-SIK1 S575 (PBL #7404) [11], and P-SIK2 S358 [34] have all been previously described.

### Plasmids

For HEK 293T overexpression studies, plasmids were used that contained the *Homo sapiens* Ubiquitin C promoter (pUbC), whose activity is unaffected by cAMP signaling: pUbC-3xFLAG-TEVsite-His_6_-MCS-IRESeGFP and pUbC-3xHA-MCS (MCS = multiple cloning site) [10].

The used plasmids code for the following *H*. *sapiens* (h) and *Mus musculus* (m) proteins (UniProt identifier): mCRTC3 (Q91X84-1), EGFP, hMARK1 TV2 (Q9P0L2-1), hMARK2 TV3 (724 amino acids; Q7KZI7-16), hMARK2 TV4 (788 amino acids; Q7KZI7-1), hMARK3 TV3 (P27448-3), hMARK4 TV2 = MARK4S (Q96L34-2), mMARK4 = MARK4L (Q8CIP4-1), hSIK1(P57059-1), mSIK2 (Q8CFH6-1), and hSIK3 (Q9Y2K2-1) (all previously described [10, 11]).

*H*. *sapiens CLASP1* isoform 2 (UniProt identifier Q7Z460-2) was obtained from the Mammalian Gene Collection (MGC; BC112940).

The *Escherichia coli* expression plasmid STII-Trx-TEVsite-mCRTC2(147–297)-His_6_ was previously described [11]. The STII-MBP-TEVsite-hTau-383-His_6_ plasmid was generated by cloning *H*. *sapiens* Tau-383 (0N4R) originating from pRK5-EGFP-Tau (Addgene #46904) into a previously described STII-MBP-TEVsite-MCS-His_6_ plasmid (STII = Strep-Tag II; MCS = multiple cloning site) [35].

MARK2 truncation constructs and plasmid backbone exchanges (3xFLAG / 3xHA) were archived by restriction cloning. Single and cumulative site-directed mutagenesis were used to introduce point mutants. The MARK2 and SIK2 hybrid proteins, the ΔMSL mutant, and the MARK2 SIK2 AL (activation loop) mutants were generated by Fusion PCR, which was performed as previously described [36].

### Cell culture

HEK293T cells were purchased from ATCC (CRL-11268) and propagated in DMEM media (Gibco®, high glucose) supplemented with 10% Fetal Bovine Serum (Gemini Bio-Products) and 100 U/ml penicillin-streptomycin (Corning Inc.).

### Overexpression & immunoprecipitation (IP)

Experiments were performed in 6 well plates by reverse transfecting HEK293T cells (2.5 × 10^6^ cells) with 2 μg plasmid DNA using Lipofectamine® 2000 (Invitrogen). 48 h post transfection cells were collected in PBS and resuspended in lysis buffer (50 mM Tris, 150 mM NaCl, 10% glycerol, 1% Igepal [Sigma-Aldrich], 1 mM DTT, EDTA-free cOmplete^™^ Protease Inhibitor Cocktail [Roche], Phosphatase Inhibitor Cocktail 2 and 3 [Sigma-Aldrich], 1 μM Carfilzomib; pH 8.0). The supernatant (= cell lysate) was either used in IP experiments or directly mixed with SDS-PAGE loading buffer. In all IP experiments, cells were pre-treated for 1 h with 1 μM Carfilzomib prior to cell lysis. Cell lysates were incubated with anti-FLAG® M2 magnetic beads and 3xFLAG peptide (100 μg/ml final) was used to elute bound proteins (both Sigma-Aldrich).

### Immunoprecipitation & mass spectrometry (IP-MS)

IP-MS of MARK2 TV4 and SIK2 AA (AA = S358A S587A) [11] was performed in 6 x 100 mm dishes by reverse transfecting HEK293T cells (1.5 × 10^7^ cells) with 12 μg plasmid DNA using Lipofectamine® 2000 (Invitrogen). 48 h post transfection cells were treated for 1h with 1 μM Carfilzomib and 10 μM Forskolin or 10 μM Doxorubicin. Cell collection and lysis was performed as described in the IP protocol.

Proteins were precipitated with 23% TCA and washed with acetone. Protein pellets were solubilized in 8 M urea, 100 mM Tris pH 8.5, reduced with 5 mM Tris(2-carboxyethyl)phosphine hydrochloride (Sigma-Aldrich), and alkylated with 55 mM 2-Chloroacetamide (Fluka Analytical). Digested proteins were analyzed by five–step MudPIT using an Agilent 1200 G1311 quaternary pump and a Thermo LTQ Orbitrap Velos using an in-house built electrospray stage [37].

Protein and peptide identification and protein quantitation were done with Integrated Proteomics Pipeline—IP2 (Integrated Proteomics Applications). Tandem mass spectra were extracted from raw files using RawConverter [38] with monoisotopic peak option. Peptide matching was done against a Uniprot *H*. *sapiens* protein database with reversed sequences and recombinant proteins using ProLuCID [39, 40] with a fixed modification of 57.02146 on C and differential modification of 79.9663 on STY. Peptide candidates were filtered using DTASelect [38, 41].

### Immunofluorescence

HEK 293T cells (0.75 × 10^6^ cells) were plated in Poly-D-Lysine coated glass bottom dishes (MatTek Corporation) and reverse transfected with Lipofectamine® 2000 (Invitrogen) using 1 μg of MARK2 and SIK2 plasmid DNA (pUbC-3xFLAG backbone with IRES GFP). 24 h post-transfection cells were fixed with 4% paraformaldehyde and incubated with primary antibodies (FLAG M2). Samples were incubated with secondary antibodies conjugated with Alexa Fluor® - 568 (goat anti-mouse) and in certain experiments counterstained with DAPI (Cayman Chemical Company) before image acquisition was performed (LSM 710; Carl Zeiss). Representative images from at least two independent experiments are shown.

### Luciferase reporter assays

The luciferase reporter plasmid contains the EVX promoter fragment (220 bp, 2x CRE half-sites) in the pGL4 backbone (Promega) [10]. Luciferase reporter assays were performed in 96 well plates by reverse transfecting HEK293T cells (100,000 cells). For each well 80 ng of DNA was used: 10 ng of EVX reporter plasmid, 10 ng of N-terminal FLAG-tagged CRTC3 plasmid, 20 ng of N-terminal FLAG-tagged MARK2/SIK2 plasmids, and 40 ng of empty pUbC-MCS-His_6_-TEVsite-3xFLAG-STOP-IRESeGFP (MCS = multiple cloning site) plasmid [10]. 24 h post transfection 10 μM Forskolin was added and cells further incubated for 4 h. DMSO served as the control treatment (each well 1% DMSO final). Next, 10 μl of Bright-Glo^™^ (Promega) was added per well and luciferase activity measured in a GloMax*®* multi microplate reader (Promega). The reporter assay was performed twice, but representative data is shown.

### *In vitro* MARK2 and SIK2 kinase assays

Expression, Ni-NTA purification, and protein concentration of STII-Trx-TEVsite-mCRTC2(147–297)-His_6_ was described previously (440 μM final) [11] and STII-MBP-TEVsite-hTau-383-His_6_ was purified in an identical manner (60 μM final). mCRTC2(147–297) contains two AMPK substrate motifs of *M*. *musculus* CRTC2 (S171 and S275) and *H*. *sapiens* Tau-383 (0N4R) harbors all four ζXKXGSXXNΨ motifs (S262, S293, S324, and S356 in hTau-441 numbering).

MARK2 (MARK2 wild type and MARK2 K205L D207A) and SIK2 (SIK2 3A [3A = S358A T484A S587A] and SIK2 3A L172K A174D) protein purification was performed in 6 x 100 mm dishes by reverse transfecting HEK293T cells (1.5 × 10^7^ cells) with 12 μg plasmid DNA using Lipofectamine® 2000 (Invitrogen). 48 h post transfection cells were treated for 1h with 1 μM Carfilzomib. Cell lysis was performed as described in the IP protocol. Following lysate incubation anti-FLAG® M2 magnetic beads were washed once in lysis buffer (50 mM Tris, 150 mM NaCl, 10% glycerol, 1% Igepal [Sigma-Aldrich], 1 mM DTT, EDTA-free cOmplete^™^ Protease Inhibitor Cocktail [Roche], Phosphatase Inhibitor Cocktail 2 and 3 [Sigma-Aldrich], 1 μM Carfilzomib; pH 8.0) and two times in buffer lacking the detergent Igepal. The identical buffer was used for elution after addition of 3xFLAG peptide (100 μg/ml final). Small aliquots of MARK2/SIK2 proteins were flash frozen in liquid nitrogen and stored at -80°C until usage. MARK2/SIK2 protein concentration was determined after SDS-PAGE and Coomassie Brilliant Blue staining (Thermo Fisher Scientific Inc.) by comparing the respective band intensities to reference bands (proteins of known concentration). Final MARK2 protein concentrations were estimated to be ~0.8 μM and SIK2 protein concentrations ~0.4 μM.

The kinase assays were performed in assay buffer (50 mM Tris, 10 mM MgCl_2_; pH 7.5) at an overall volume of 80 μl. mCRTC2(147–297) and hTau-383 proteins were pre-diluted in assay buffer to 40 μM. To start the reaction components were sequentially added to pre-chilled assay buffer (incubation on ice): 1 μl of mCRTC2(147–297) (0.5 μM final), 1 μl of hTau-383 (0.5 μM final), 0.8 μl of 1 mM ATP solution (10 μM final; Sigma-Aldrich), and 0.025 μM MARK2/SIK2 proteins (~2.5–5 μl). The reaction tubes were vortexed, spun down, and incubated at 30°C in a ThermoMixer® R (Eppendorf AG). At the indicated time points 10 μl of the reaction mixture was removed and boiled after addition of SDS sample buffer (supplemented with 10 mM EDTA).

### Sequence alignment

Amino acid sequences were aligned using MegAlign and Clustal W method (DNASTAR v7).

Protein sequences were obtained from UniProt [42], with the following UniProt identifiers: MARK1-4 (Q9P0L2-1, Q7KZI7-1, P27448-5, MARK4L Q96L34-1), SIK1-3 (P57059-1, Q9H0K1-1, Q9Y2K2-1), BRSK1,2 (Q8TDC3-1, Q8IWQ3-1), PRKAA1,2 (Q13131-1, P54646-1), NUAK1,2 (O60285-1, Q9H093-1), and SNRK (Q9NRH2-1).

### Visualization of protein structures

The data files of MARK2 structures were obtained from the Protein Data Bank (PDB ID: 3IEC [43] & 2WZJ [44]) and modified/visualized using PyMol V1.3 (Schrödinger).

### Statistical analysis

Data are either presented as the mean ± SEM. or ± SD. Statistical analysis was performed using Microsoft Excel (Microsoft Corporation) or PRISM (GraphPad) and graphical presentations were generated using SigmaPlot (Systat Software Inc.) or PRISM.

## Results

### Comparison of MARK2 and SIK2 activities on cellular ζXKXGSXXNΨ motif phosphorylation

**Fig 1:**
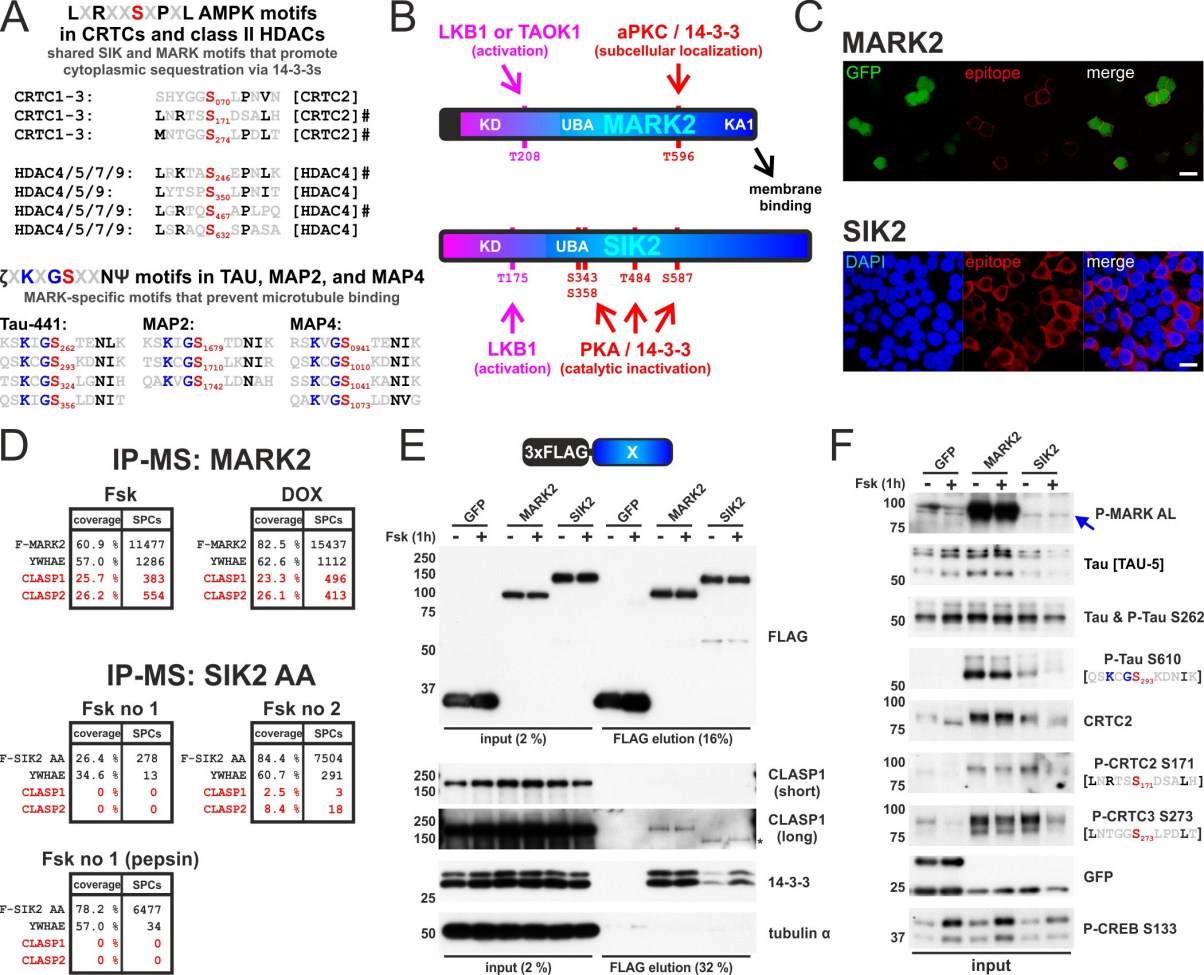
Comparison of MARK2 and SIK2 reveals differences in ζXKXGSXXNΨ motif phosphorylation and interactions with CLASP proteins. A) Confirmed and putative phosphorylation sites of MARKs and SIKs [9–13, 45]: Common AMPK family substrate motifs in cAMP-regulated transcriptional co-activators (CRTCs: CRTC1-3) and class IIA Histone deacetylases (HDACs: HDAC4,5,7,9) as well as ζXKXGSXXNΨ motifs in tau (numberings are according to *Homo sapiens* CRTC/HDAC proteins, Tau-441, and isoform 1 of MAP2/4). # indicates motifs that were previously shown to be phosphorylated by both MARKs and SIKs. B) To scale schematic depiction of *H*. *sapiens* MAP/microtubule affinity-regulating kinase 2 (MARK2 1–788) and salt-inducible kinase 2 (SIK2 1–926). MARK2 and SIK2 share homology inside their N-terminal kinase domain (KD) and are both activated by LKB1, but encode distinct C-termini. C) Representative immunofluorescence image of HEK 293T cells transfected with FLAG-tagged MARK2 and SIK2, which were expressed from the constitutive Ubiquitin C promoter (UbC). These plasmids coexpress EGFP via an internal ribosome entry site (IRES). Cells were stained for the FLAG epitope and either imaged for EGFP (MARK2) or counterstained with DAPI (SIK2). (scale bar indicates 20 μm). D) Tables show the recovery of N-terminally FLAG-tagged MARK2 (F-MARK2; hMARK2 TV4) and SIK2 (F-SIK2 AA, with AA = S358A S587A) subjected to mass spectrometry (MS) analysis following the co-IP (SPCs = spectral counts). Recovery of endogenous 14-3-3 ε (YWHAE) and CLIP-associating proteins (CLASP1/2) are indicated. Samples were either treated with the adenylyl cyclase activator Forskolin (Fsk; 10 μM for 1h) or the DNA-damaging agent Doxorubicin (DOX, 10 μM for 4h) prior to cell lysis and IP-MS protocol. In case of SIK2 AA one sample was not only subjected to trypsin but also to pepsin proteolytic digest. E) Western blot analysis of the co-IP of FLAG-tagged GFP, MARK2, and SIK2 with endogenous CLASP1 and 14-3-3 proteins. Prior to the co-IP, cells were treated for 1 h with either DMSO or 10 μM Fsk for 1h. F) Western blot analysis of the corresponding input, resolving MARK activation loop phosphorylation (P-MARK AL; blue arrow indicates endogenous HEK 293T cell signal at ~ 85 kDa) as well as phosphorylation of AMPK and ζXKXGSXXNΨ motifs inside CRTCs and tau. EGFP is coexpressed via an IRES from all expression plasmids.

The closely related SIK and MARK families phosphorylate conventional AMPK substrate motifs within CRTCs (CRTC1-3) and class IIA HDACs (HDAC4,5,7,9) (Fig 1A). By contrast, phosphorylation of tau and MAPs at KXGS motifs appears to be more MARK-selective [19]. Closer inspection of the KXGS motifs in tau and MAP2/4 reveals that all KXGS sites contain a Asn at position +3, and 10 of the 11 sites have a hydrophobic residue at position +4 (Ψ = I, L, V). In contrast to the LXRXXSXXXL AMPK consensus, the KXGS motifs have a hydrophilic residue (ζ = Q, T, S, R, K) in place of the characteristic hydrophobic residue at position -5, resulting in a refined ζXKXGSXXNΨ motif.

Recent studies have implicated other AMPK family members, including the SIKs [30], in the phosphorylation of these variant ζXKXGSXXNΨ sites. SIKs and MARKs show considerable sequence homology within the N-terminal kinase and UBA domains; and both kinase families are activated by LKB1 phosphorylation (Fig 1B). By contrast, the C-terminus of SIK2 enables inhibition by cAMP/PKA while the C-terminus of MARK2 mediates KA1-dependent and aPKC-regulated membrane localization (Fig 1B and 1C). Based on these properties, we considered that MARK-specific regions might modulate ζXKXGSXXNΨ-directed specificity by bringing tau and MAP2/4 substrates into physical proximity with the catalytic domain. To test this notion, we expressed full-length FLAG-tagged MARK2 and SIK2 in HEK 293T cells and evaluated protein-protein interactions by immunoprecipitation-mass spectrometry (IP-MS) (Fig 1D). Across all experiments, only MARK2 showed strong interactions with CLIP-associating proteins 1 and 2 (CLASP1/2), a family of microtubule-binding proteins [46]. We confirmed this result by co-immunoprecipitation (co-IP) of cells overexpressing MARK2 or SIK2, followed by detection of endogenous CLASP1 using Western blot analysis (Fig 1E).

The adenylyl cyclase activator Forskolin (Fsk) stimulates cAMP-mediated induction of PKA, leading to S133 phosphorylation and activation of the transcription factor cAMP response-element binding protein (CREB) [47]. In parallel, PKA also phosphorylates and inactivates the SIKs by promoting 14-3-3 binding, leading to dephosphorylation and nuclear translocation of the CRTC family of CREB coactivators [48]. Correspondingly, exposure to Fsk promoted the interaction of SIK2 with 14-3-3 proteins, thereby reducing CRTC phosphorylation at AMPK substrate motifs (P-CRTC2 S171 and P-CRTC3 S273 antibodies) (Fig 1E and 1F). Fsk had no discernable effect on MARK2 interactions with 14-3-3s and CLASP1 nor on MARK-dependent phosphorylation of CRTCs [10]. Overexpression of either MARK2 or SIK2 promoted hyperphosphorylation of endogenous ζXKXGSXXNΨ motifs inside tau (P-Tau S610 antibody = phospho-tau S293 in Tau-441, Fig 1F). However, MARK2 overexpression increased tau phosphorylation by more than 2-fold in comparison with SIK2 overexpression, confirming that MARK2 has higher activity against ζXKXGSXXNΨ motifs.

**Fig 2:**
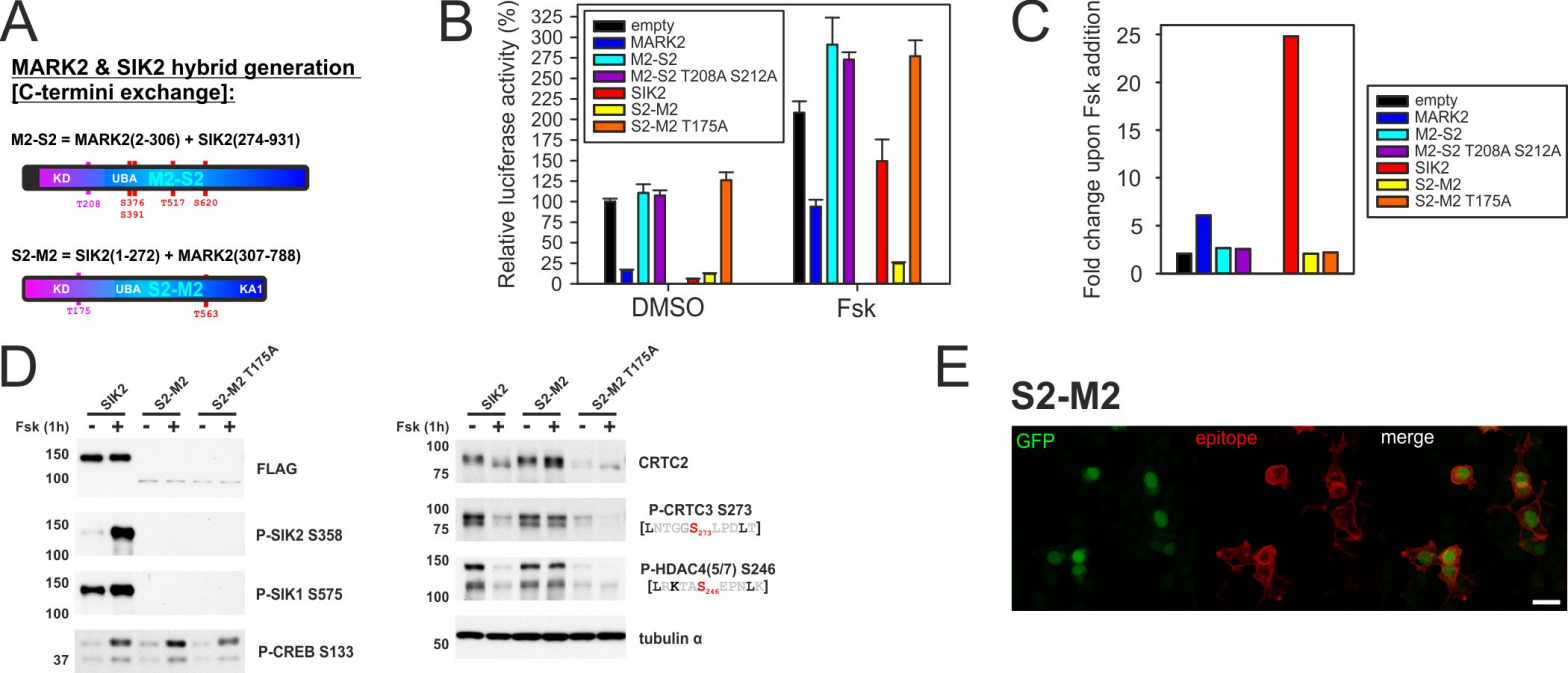
The C-terminus of SIK2 confers cAMP responsiveness. A) To scale schematic depiction of MARK2 (M2) and SIK2 (S2) hybrid proteins that were evaluated for catalytic activity. M2-S2 encodes the N-terminal kinase domain of MARK2 with the C-terminus of SIK2 (4x PKA sites) and S2-M2 the kinase domain of SIK2 with the C-terminus of MARK2 (1x aPKC site). B) EVX-Luc reporter assay measuring the effect of MARK2 and SIK2 hybrid proteins as well as activation loop mutants (MARK2/SIK2 = T208A/S175A) upon the transcriptional activity of coexpressed CRTC3. EVX-Luc activity was measured after 4 h of DMSO/Fsk treatment. (n = 5, ± SEM) C) Graph depicting the corresponding fold changes in reporter activity upon 10 μM Fsk treatment. D) Western blot analysis showing the effects of overexpressed FLAG-tagged SIK2 wild type, S2-M2 hybrid protein, and S2-M2 activation loop mutant (T175A) on the phosphorylation of endogenous CRTCs and class IIA HDACs. (DMSO and 10 μM Fsk treatment for 1h) E) Representative immunofluorescence image of HEK 293T cells transfected with FLAG-tagged S2-M2 hybrid protein. Cells were stained for the FLAG epitope and imaged for EGFP, which is coexpressed via an IRES from the S2-M2 expression plasmid. (scale bar indicates 20 μm).

Regulatory differences of MARKs and SIKs are encoded by their divergent C-terminal sequences, which dictate membrane localization and cAMP sensitivity, respectively [11, 14]. To determine the impact of these regions on catalytic activity, we generated hybrid proteins exchanging the regulatory domains of MARK2 and SIK2 (Fig 2A). We employed a cyclic*-*AMP response element (CRE)-based reporter assay to evaluate the repressive activity of these MARK2/SIK2 hybrid proteins on CRTC-dependent luciferase gene expression [49] (Fig 2B and 2C). The MARK2 kinase domain was inactive upon fusion with the C-terminus of SIK2 (M2-S2), because neither M2-S2 nor its respective activation loop mutant (T208A S212A [50]) had any effect on CRE activity. In contrast, the SIK2 kinase domain remained active following fusion to the C-terminus of MARK2 (S2-M2), where it efficiently repressed CRE reporter activity. Consistent with our previous results that C-terminal phosphorylation by PKA and 14-3-3 binding inhibits SIK2 catalytic activity [11], the S2-M2 hybrid was active in repressing CRE reporter activity even upon Fsk stimulation. We confirmed that exposure to Fsk increased SIK2 phosphorylation at PKA sites S358 and S587 (Fig 2D) [11, 34]. Consistent with its inhibitory effect on wild type SIK2, Fsk promoted dephosphorylation of CRTCs and class IIA HDACs. Although the S2-M2 hybrid protein was destabilized in comparison to SIK2 and had weaker membrane localization relative to MARK2 (Fig 2E), S2-M2 phosphorylated CRTC/HDAC in a Fsk-resistant manner (Fig 2D). Taken together, these results indicate that SIK2 and MARK2 are distinguished by their relative sensitivity to cAMP, subcellular localization, and interaction with CLASP1/2 proteins. These differences may contribute to the increased cellular potency of MARK2 in phosphorylating variant ζXKXGSXXNΨ motifs in tau and other substrates.

### MARK1-3 interact with the microtubule-binding CLASP1 protein via their C-terminal KA1 domain

**Fig 3:**
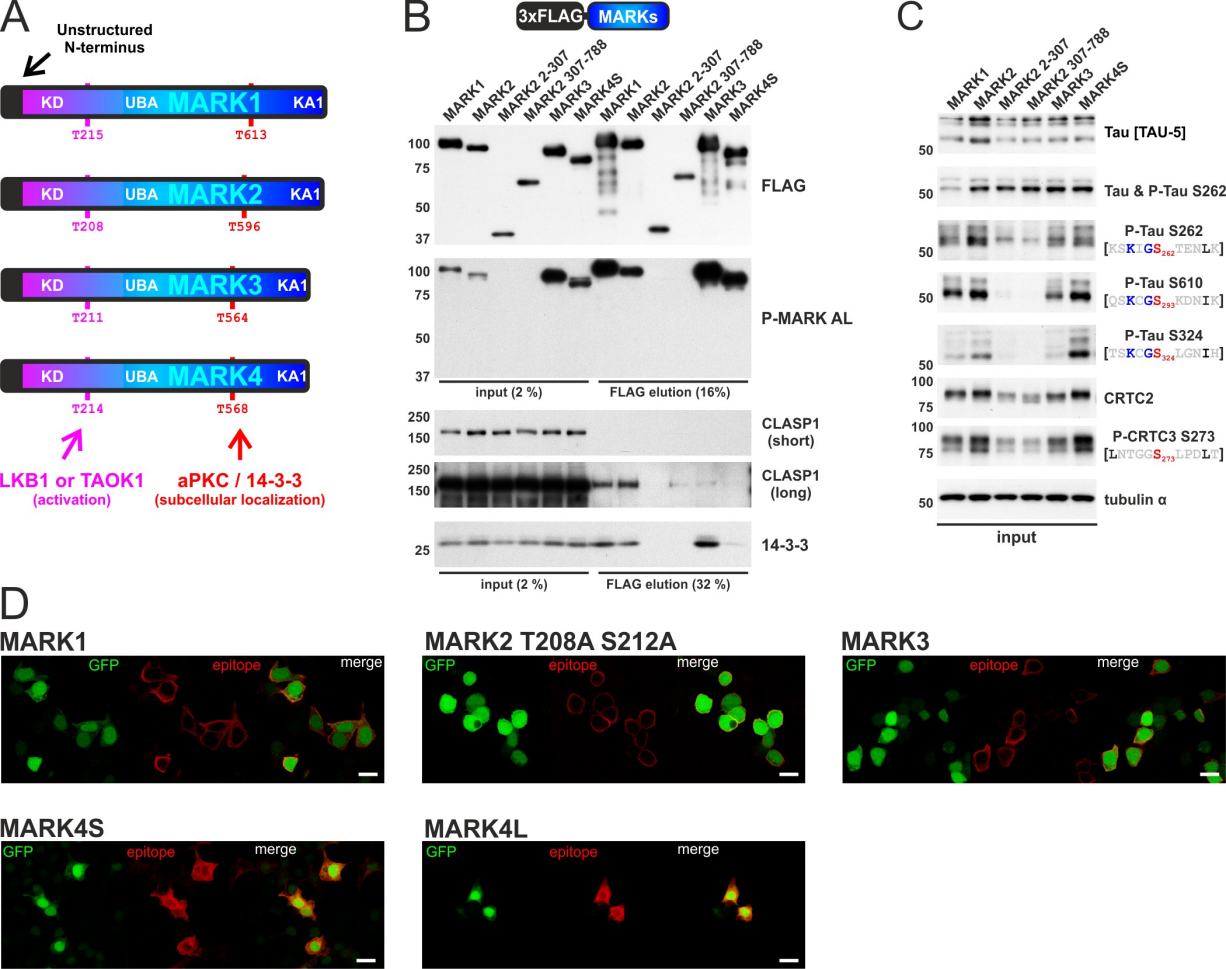
The MARK family displays distinct CLASP1 interactions and subcellular localizations. A) To scale schematic depiction of *H*. *sapiens* MARK family (MARK1-4). At the N-terminus all MARKs contain an unstructured region, followed by the kinase domain (KD), the ubiquitin-associated domain (UBA), a variable regulatory domain, and at the C-terminus the kinase associated 1 (KA1) domain. Phosphorylation at a conserved threonine inside the activation loop by either LKB1 or TAOK1 activates MARKs and phosphorylation within the regulatory domain controls 14-3-3 binding and subcellular localization (among other kinases by aPKC). B) Western blot analysis of the co-IP of FLAG-tagged MARK1-4 with endogenous CLASP1 and 14-3-3 proteins. Additionally, two truncation constructs of MARK2 were assayed: The N-terminal kinase domain of MARK2 (MARK2 1–307) and the C-terminus of MARK2, which starts at the UBA domain (MARK2 307–788). C) Western blot analysis of the corresponding input, resolving AMPK and ζXKXGSXXNΨ motif phosphorylation inside endogenous CRTCs and tau. D) Representative immunofluorescence image of HEK 293T cells transfected with FLAG-tagged MARK1, the inactive MARK2 T208A S212A activation loop mutant, MARK3, and both MARK4 isoforms (MARK4S and MARK4L) that differ at their C-termini [42]. Cells were stained for the FLAG epitope and imaged for EGFP, which is coexpressed via an IRES from all expression plasmids. (scale bar indicates 20 μm).

The MARK family consists of four members, whose activities are modulated by LKB1/TAOK1- and aPKC-mediated phosphorylation (Fig 3A). MARKs also harbor a ~100 amino acid conserved KA1 domain, which typically mediates membrane localization [14]. To investigate the relationship between CLASP1 interaction, subcellular localization, and ζXKXGSXXNΨ motif phosphorylation, we performed overexpression studies with members of the MARK family (MARK1-4) (Fig 3B and 3C). In co-IPs, MARK1-3 associated with CLASP1 and MARK1 and MARK2 showed stronger interactions with CLASP1 than MARK3 (Fig 3B). Out of two non-overlapping truncation mutants of MARK2 (2–307 and 307–788), only the C-terminal domain (MARK2 307–788) bound CLASP1. All four MARKs mediated efficient phosphorylation of both ζXKXGSXXNΨ motifs inside tau (sites S262, S293, and S324) as well as the AMPK motifs in the CRTCs (Fig 3C), with MARK2/4 appearing more potent than MARK1/3. Consistent with the importance of the UBA domain for LKB1-mediated MARK activation (T208 in MARK2) [5], MARK2 2–307 containing only the kinase domain was not phosphorylated within the activation loop (AL; P-MARK AL antibody) and consequently, was unable to phosphorylate cellular substrates (Fig 3B and 3C). In immunofluorescence studies of HEK 293T cells, we confirmed the association of MARK1-3 with cell membranes [14] and the interaction of MARK4 with microtubules and microtubule organizing centers (MTOCs) [51] (Fig 3D). MARK2 showed the strongest membrane binding, which was unaffected by the catalytically inactivating Thr to Ala activation loop mutant (MARK2 T208A S212A).

**Fig 4:**
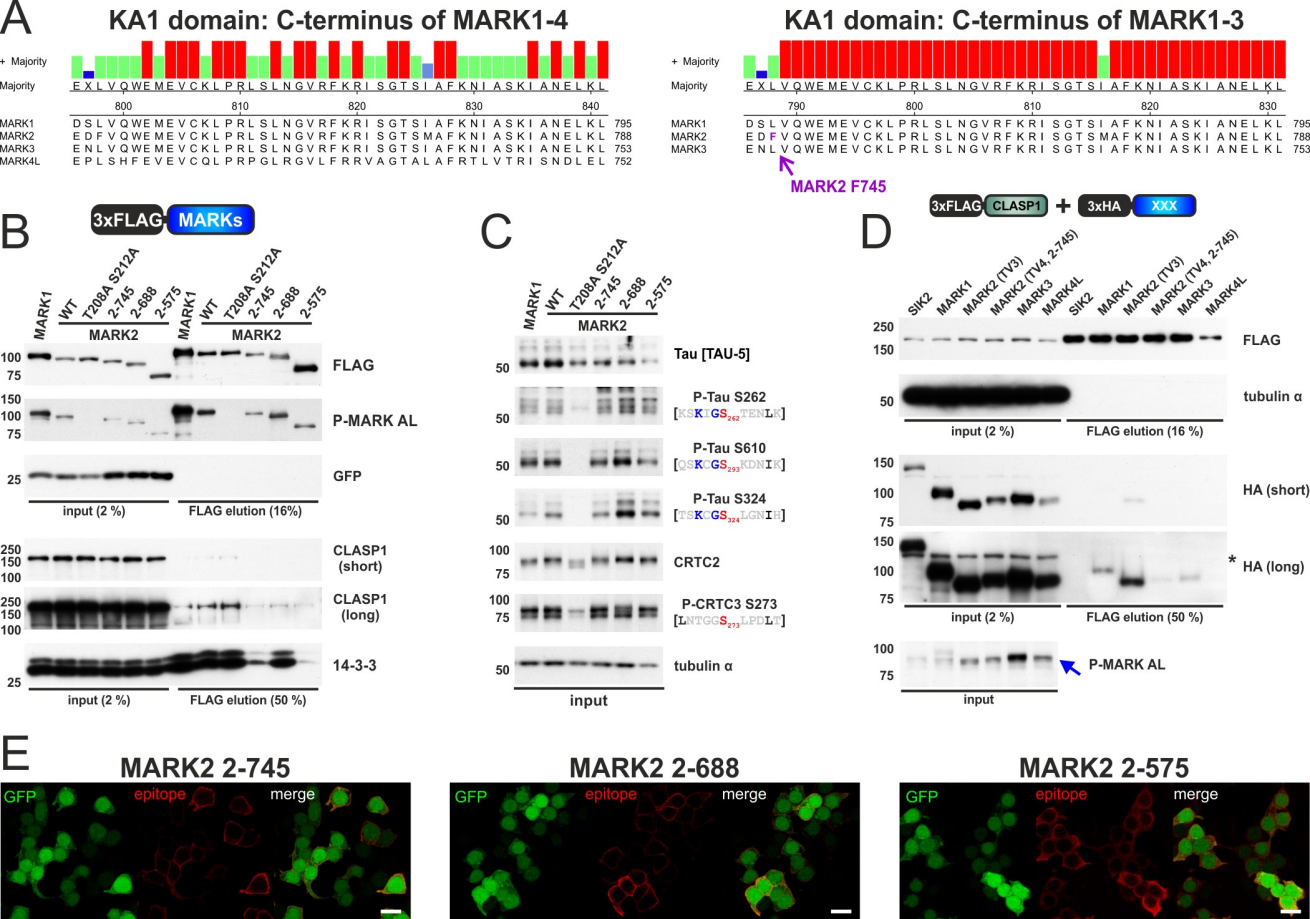
The KA1 domain influences CLASP1 interaction and plasma membrane localization. A) On the left, the sequence alignment of the C-terminus of MARK1-4, which is part of the ~100 amino acid KA1 domain. On the right, the sequence alignment of the C-terminus of MARK1-3. The truncation point for the MARK2 2–745 mutant is indicated (MARK2 F745). B) Western blot analysis of the co-IP of FLAG-tagged MARK1 as well as MARK2 wild type (WT) and respective mutants with endogenous CLASP1 and 14-3-3 proteins. EGFP is coexpressed via an IRES from all expression plasmids. C) Western blot analysis of the corresponding input, resolving AMPK motif and ζXKXGSXXNΨ motif phosphorylation inside endogenous CRTCs and tau. D) Western blot analysis of the co-IP of FLAG-tagged CLASP1 with HA-tagged SIK2, MARK1-4, and the MARK2 2–745 truncation mutant. For the HA-tagged MARK2 construct transcript variant 3 was used (TV3: MARK2 1–724; UniProt Q7KZI7-4), while the MARK2 2–745 mutant was generated in transcript variant 4 background (UniProt Q7KZI7-1). The P-MARK AL antibody detects the activation loop phosphorylation of overexpressed MARK1-4 as well as an endogenous HEK 293T cell signal at ~ 85 kDa (see blue arrow). E) Representative immunofluorescence image of HEK 293T cells transfected with consecutive C-terminal truncation mutants of FLAG-tagged MARK2. Cells were stained for the FLAG epitope and imaged for EGFP, which is coexpressed via an IRES from the expression plasmids. (scale bar indicates 20 μm).

Although the interaction with the microtubule-associating CLASP1 proteins was limited to MARK1-3, all MARKs hyperphosphorylate ζXKXGSXXNΨ motifs on cellular tau. MARK4 on the other hand binds by itself to microtubules and might therefore not require CLASP1 interaction for proximity-dependent ζXKXGSXXNΨ phosphorylation. Sequence comparison of MARK1-4 revealed that the C-terminal KA1 domain is highly conserved among MARK1-3 and more variable or even absent in MARK4 isoforms [42] (Fig 4A). To further evaluate the effect of CLASP1 binding for ζXKXGSXXNΨ motif phosphorylation, we generated consecutive C-terminal truncations of MARK2 (Fig 4B and 4C). Deletion of its extreme C-terminus (MARK2 2–745) severely impaired the binding of MARK2 to endogenous CLASP1 (Fig 4B). As MARK2 2–688 and 2–575 mutants had no further inhibitory effect on CLASP1 binding relative to MARK2 2–745, we conclude that the distal 45 amino acids of the KA1 domain are required for complex formation. Despite the loss of CLASP1 binding, MARK2 mutants were still capable of phosphorylating ζXKXGSXXNΨ and AMPK motifs comparably (Fig 4C). The MARK2-CLASP1 interaction was subsequently confirmed by coexpression of FLAG-tagged CLASP1 and HA-tagged MARK1-4 (Fig 4D). Consistent with the observed interaction patterns for endogenous CLASP1, overexpressed CLASP1 bound most strongly to wild type MARK2 relative to MARK1/3 and the MARK2 2–745 mutant showed dramatically impaired complex formation. Since the KA1 domain has previously been implicated in membrane binding [14], we studied our C-terminal truncation mutants by immunofluorescence (Fig 4E). Although MARK2 2–745 still bound predominantly to the plasma membrane, the MARK2 2–575 mutant localized completely inside the cytoplasm. Thus, although MARK family members exhibit distinct subcellular localization and interactions with the microtubule-associating protein CLASP1, these associations have no detectable effect on catalytic activity or ζXKXGSXXNΨ motif phosphorylation.

### The kinase domain of MARK2 mediates efficient ζXKXGSXXNΨ motif phosphorylation

**Fig 5:**
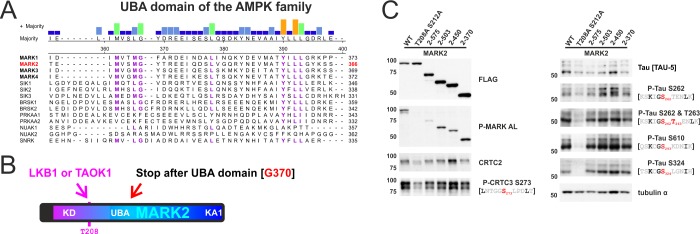
The N-terminus of MARK2 is sufficient to phosphorylate ζXKXGSXXNΨ motifs. A) Sequence alignment of the ubiquitin-associated (UBA) domain of the AMPK family, which plays a role for most kinases in mediating inactions with LKB1. Although LKB1 can activate AMPK (PRKAA1/2) and NUAK1/2[3], these kinases lack key features of the UBA domain (highlighted in purple) [5]. B) To scale schematic depiction of *H*. *sapiens* MARK2 (MARK2 1–788; UniProt identifier Q7KZI7-1) with the truncation point after the UBA domain indicated (G370). C) Western blot analysis of the overexpression of FLAG-tagged MARK2 truncation mutants and their effect upon AMPK and ζXKXGSXXNΨ motif phosphorylation inside endogenous CRTCs and tau.

Realizing that the C-terminal KA1 domain is dispensable for MARK2-mediated ζXKXGSXXNΨ phosphorylation, we evaluated effects of other MARK2 regions on catalytic activity. MARK family members contain a UBA domain, which is necessary for LKB1-mediated activation inside cells [5] (Fig 5A). Consequently, the smallest active mutant (MARK2 2–370) contained the catalytic and UBA domains (Fig 5B and 5C). All three MARK2 truncations were phosphorylated at their activation loop and had comparable activity on both AMPK and ζXKXGSXXNΨ substrate motifs. These results indicate that a protein fragment containing kinase and UBA domains of MARK2 (MARK2 2–370) is sufficient for cellular phosphorylation of ζXKXGSXXNΨ motifs.

**Fig 6:**
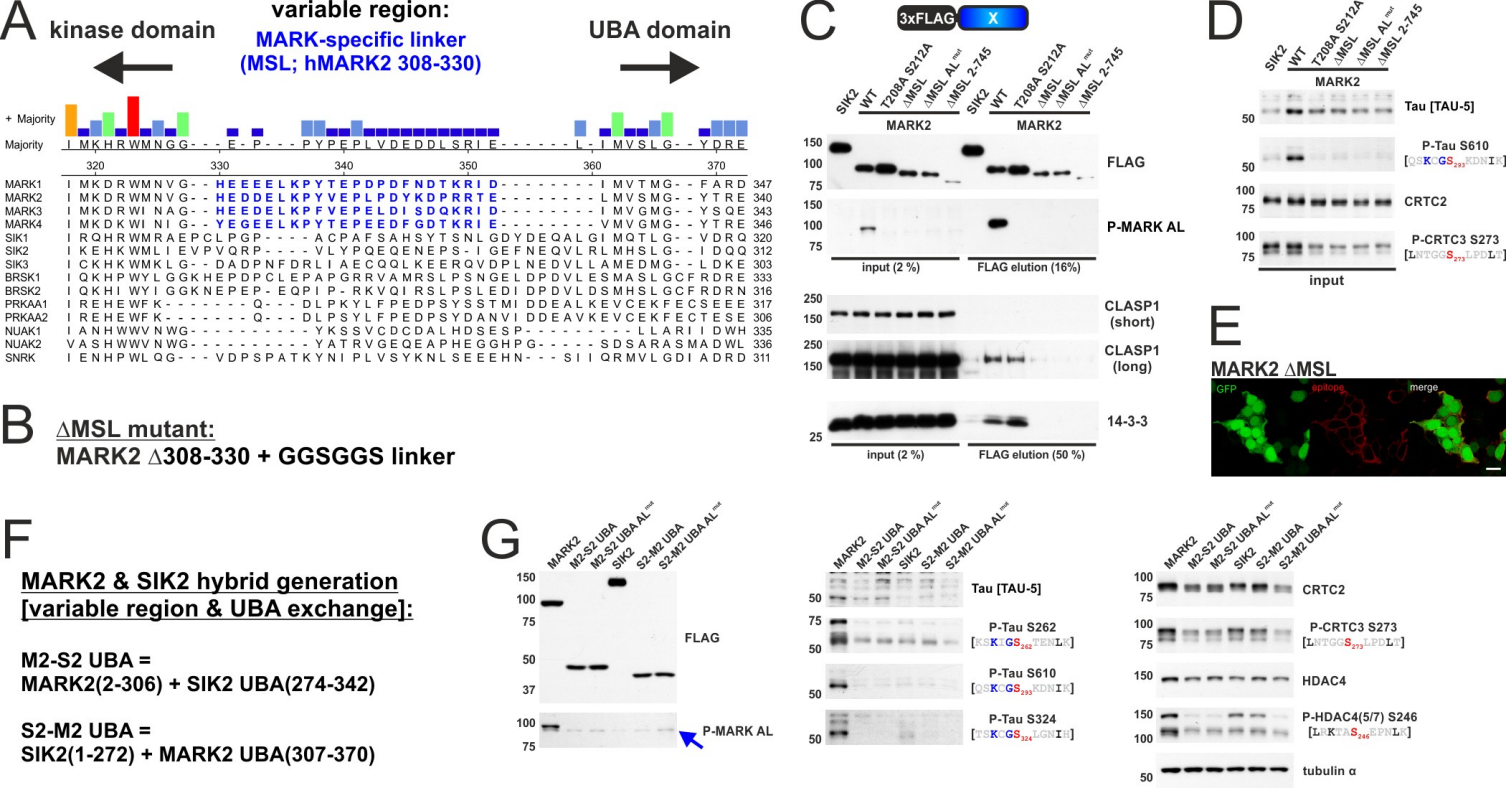
The MARK-specific linker (hMARK2 308–330) is required for kinase activity, but does not influence ζXKXGSXXNΨ motif phosphorylation. A) Sequence alignment of a variable linker region framed by the N-terminal kinase domain and the C-terminal ubiquitin-associated (UBA) domain of the AMPK family. This linker region is conserved across MARK1-4 and therefore termed MARK-specific linker (MSL, hMARK2 308–330, highlighted in blue). B) The ΔMSL mutant was generated by replacing hMARK2 308–330 by a GGSGGS flexible linker. C) Western blot analysis of the co-IP of FLAG-tagged SIK2, MARK2, and ΔMSL mutants with endogenous CLASP1 and 14-3-3 proteins. (AL^mut^ = T208A S212A) D) Western blot analysis of the corresponding input, resolving AMPK and ζXKXGSXXNΨ motif phosphorylation inside endogenous CRTCs and tau. E) Representative immunofluorescence image of HEK 293T cells transfected with FLAG-tagged MARK2 ΔMSL. Cells were stained for the FLAG epitope and imaged for EGFP, which is coexpressed via an IRES from the expression plasmids. (scale bar indicates 20 μm). F) Amino acid sequence of MARK2 (M2) and SIK2 (S2) hybrid proteins that were evaluated for catalytic activity. M2-S2 UBA encodes the N-terminal kinase domain of MARK2 with the variable region & UBA domain of SIK2. S2-M2 UBA consists of the kinase domain of SIK2 with the MSL & UBA domain of MARK2. G) Western blot analysis of the overexpression of FLAG-tagged MARK2, SIK2, and respective hybrid proteins. The effect upon AMPK and ζXKXGSXXNΨ motif phosphorylation was determined inside endogenous CRTCs, class IIA HDACs, and tau. The blue arrow indicates the P-MARK AL signal from endogenous HEK 293T cell signal at ~ 85 kDa.

Sequence comparison of MARKs (MARK1-4) with the other AMPK family members revealed a 22 amino acid MARK-specific linker (MSL; MARK2 amino acids 308–330) (Fig 6A), which is located between the N-terminal kinase and C-terminal UBA domains. Replacement of the MSL with a flexible GGSGGS linker (ΔMSL; Fig 6B) disrupted MARK catalytic activity as well as CLASP1 and 14-3-3 binding (Fig 6C and 6D). Because these interactions are mediated by sequences outside the MSL, we hypothesize that loss of this region must disrupt protein folding. Nevertheless, ΔMSL still bound predominantly to membranes (Fig 6E), supporting the notion that the KA1 domain is partially functional.

We generated MARK2 and SIK2 hybrid proteins to further define the role of the MSL for ζXKXGSXXNΨ-directed MARK2 activity (Fig 6F). M2-S2 UBA carries the kinase domain of MARK2 with the linker sequence and UBA domain of SIK2, while S2-M2 UBA encodes the kinase domain of SIK2 with the MSL and UBA domain of MARK2 (Fig 6G). The kinase domain of MARK2 was not functional in the context of SIK2-derived sequences (M2-S2 UBA versus activation loop mutant); but the kinase domain of SIK2 was active following fusion with the MSL and UBA domain of MARK2 (S2-M2 UBA). However, S2-M2 UBA did not increase ζXKXGSXXNΨ motif phosphorylation relative to SIK2 wild type, indicating that the MSL and UBA domains of MARK2 do not influence ζXKXGSXXNΨ-directed activity. Taken together, these results indicate that, although the presence of the MARK-specific linker (MSL) and UBA domain are absolutely required for cellular activity, phosphorylation of the variant ζXKXGSXXNΨ motif is mediated by the kinase domain of MARK2 (amino acids 2–306).

### The role of the KLDpT motif inside MARKs activation loop

**Fig 7:**
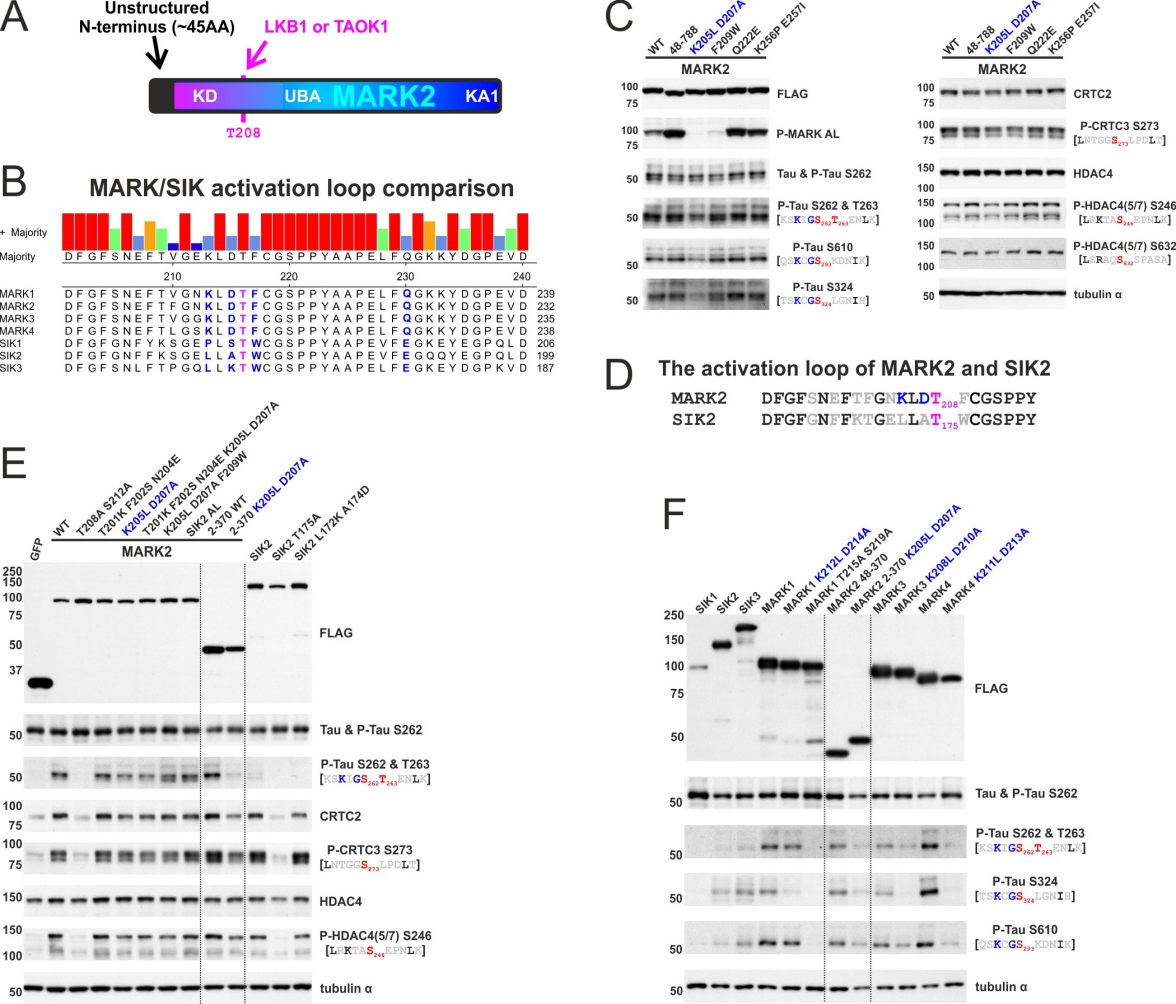
Cellular MARK activity is impaired by mutation of the KLDpT208 activation loop motif. A) To scale schematic depiction of *H*. *sapiens* MARK2 (MARK2 1–788). Highlighted are the unstructured N-terminus (~ amino acids 1–45) and the activation loop threonine, which is phosphorylated by LKB1/TAOK1 and required for cellular activity. B) Sequence alignment of the activation loop of MARKs and SIKs. C) Western blot analysis of the overexpression of FLAG-tagged MARK2, the N-terminal truncation mutant (MARK2 48–788), and various point mutants. The effect upon AMPK and ζXKXGSXXNΨ motif phosphorylation was determined inside endogenous CRTCs, class IIA HDACs, and tau. D) Sequence comparison of the *H*. *sapiens* MARK2 and *M*. *musculus* SIK2 activation loops, which differ in 8 residues. The KLDpT motif is highlighted in blue and additional differences in grey. E) Western blot analysis of the overexpression of FLAG-tagged GFP, SIK2, MARK2, and activation loop mutants. The KLDpT208 motif is present is MARK2, while LLApT175 is found in SIK2. The MARK2 SIK2 AL mutant carries the complete SIK2 activation loop (8x mutant). The effect upon AMPK and ζXKXGSXXNΨ motif phosphorylation was determined inside endogenous CRTCs, class IIA HDACs, and tau. Blots were overlaid with dotted lines to facilitate comparison of MARK2 2–370 wild type (WT) and K205L D207L. F) Western blot analysis of the overexpression of FLAG-tagged SIK1-3 as well as MARK1-4 with the respective LLApT activation loop mutants, resolving ζXKXGSXXNΨ motif phosphorylation inside endogenous tau. Blots were overlaid with dotted lines to facilitate comparison of MARK2 48–370 WT and MARK2 2–370 K205L D207L.

Having seen that the kinase domain of MARKs has higher ζXKXGSXXNΨ-directed activity relative to SIKs, we examined the sequences within the kinase domain of MARK1-4 and SIK family members. We noticed that a number of MARK2 isoforms lack the unstructured N-terminus [42] (Fig 7A). Around the activation loop, MARK1-4 also contain residues that are conserved within this subfamily, but that differ in SIK family members (Fig 7B). In turn, we examined effects of deleting the unstructured N-terminus or mutating residues inside the activation loop of MARK2 to corresponding residues in SIK2 (Fig 7C).

Deletion of the unstructured N-terminus (MARK2 48–788) had no effect on AMPK and ζXKXGSXXNΨ motif phosphorylation. But an activation loop mutant of MARK2 (MARK2 K205L D207A) was more strongly impaired in its ability to phosphorylate tau at ζXKXGSXXNΨ sites S262, S293, and S324 than at AMPK motifs inside CRTCs and class IIA HDACs. Although the activation loops of MARK2 and SIK2 differ at eight residues (Fig 7D), mutation at any of these residues did not further disrupt MARK2 catalytic activity relative to MARK2 K205L D207A (Fig 7E). However, replacement of the complete activation loop with the SIK2 sequence (MARK2 SIK2 AL) rescued cellular activity against ζXKXGSXXNΨ and AMPK motifs. The impairment of MARK2 activity by K205L D207A was also evident within the stand-alone kinase domain fragment (MARK2 2–370), confirming that the motif-specific catalytic inhibition is independent of the regulatory C-terminus. In contrast to MARK2, the corresponding activation loop mutant inside SIK2 (L172K A174D) affected neither ζXKXGSXXNΨ nor AMPK motif phosphorylation. As the KLDpT to LLApT activation loop mutants in MARK1/3/4 also impaired ζXKXGSXXNΨ phosphorylation (Fig 7F), these results support the notion that the molecular mechanism underlying this process is MARK-specific and conserved.

**Fig 8:**
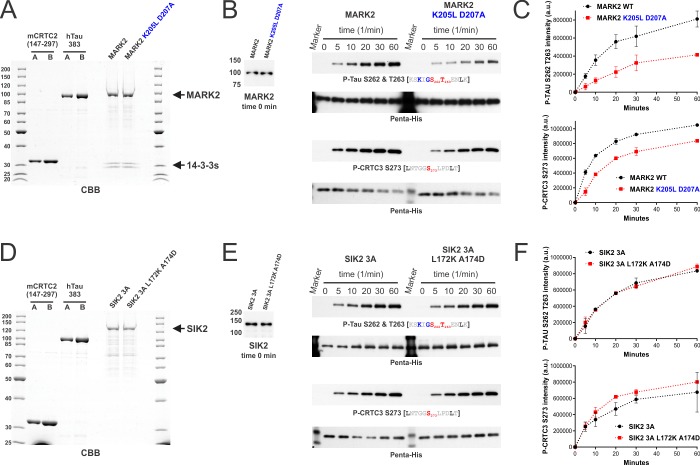
The KLDpT motif modulates MARK2 activity *in vitro*. A) Coomassie brilliant blue (CBB) stained SDS-PAGE of heterologously expressed and His-tag purified CRTC2 fragment (STII-Trx-TEVsite-mCRTC2(147–297)-His_6_) and full-length tau (STII-MBP-TEVsite-hTau-383-His_6_) (A/B = 0.5/1 μg loaded protein amount). FLAG-MARK2 wild type and K205L D207A were overexpressed in HEK 293T cells and proteins subsequently purified by co-IP and FLAG peptide elution (~0.6 μg loaded protein amount). B) Western blot analysis of the time-dependent *in vitro* kinase assay of MARK2. CRTC2 and tau served as substrates within the same assay. See materials and methods for details. At the 0-minute time point, the amount of MARK2 was determined via a MARK2-specific antibody. C) Quantification of the phosphorylation of tau (P-Tau S262 T263 antibody; n = 3, ±SD) and CRTC2 (P-CRTC3 S273 antibody; n = 2, ±SD) by densitometric analysis. D) Coomassie brilliant blue (CBB) stained SDS-PAGE of heterologously expressed and His-tag purified CRTC2 fragment (STII-Trx-TEVsite-mCRTC2(147–297)-His_6_) and full-length tau (STII-MBP-TEVsite-hTau-383-His_6_) (A/B = 0.5/1 μg loaded protein amount). FLAG-SIK2 3A (3A = S358A T484A S587A) and L172K A174D were overexpressed in HEK 293T cells and proteins subsequently purified by co-IP and FLAG peptide elution (~0.25 μg loaded protein amount). E) Western blot analysis of the time-dependent *in vitro* kinase assay of SIK2. CRTC2 and tau served as substrates within the same assay. See materials and methods for details. At the 0-minute time point, the amount of SIK2 was determined via a SIK2-specific antibody. F) Quantification of the phosphorylation of tau (P-Tau S262 T263 antibody) and CRTC2 (P-CRTC3 S273 antibody) by densitometric analysis (n = 2, ±SD).

We purified the full-length proteins of MARK2, SIK2, and their respective activation loop mutants from HEK 293T cells to determine whether the KLDpT208 motif is also required for *in vitro* activity of MARK2 (Fig 8). To obtain appropriate unphosphorylated ζXKXGSXXNΨ and AMPK substrates, we purified tau (Tau-383/0N4R) and a CRTC2 fragment (147–297 of mCRTC2) following their heterologous expression in *Escherichia coli*. In the corresponding *in vitro* kinase assay, we quantified the parallel phosphorylation of S274 AMPK motif inside CRTC2 (P-CRTC3 S273) and S262 ζXKXGSXXNΨ motif inside tau (P-Tau S262 T263). Consistent with the cellular data, the K205L D207A mutant within the MARK2 activation loop disrupted the *in vitro* phosphorylation of ζXKXGSXXNΨ and AMPK motifs (Fig 8A–8C). In contrast, the L172K A174D mutant had no detectable effect on the *in vitro* activity of SIK2 (Fig 8D–8F). Collectively, these results demonstrate that the KLDpT208 activation loop motif promotes catalytic activity of MARK2.

## Discussion

**Fig 9:**
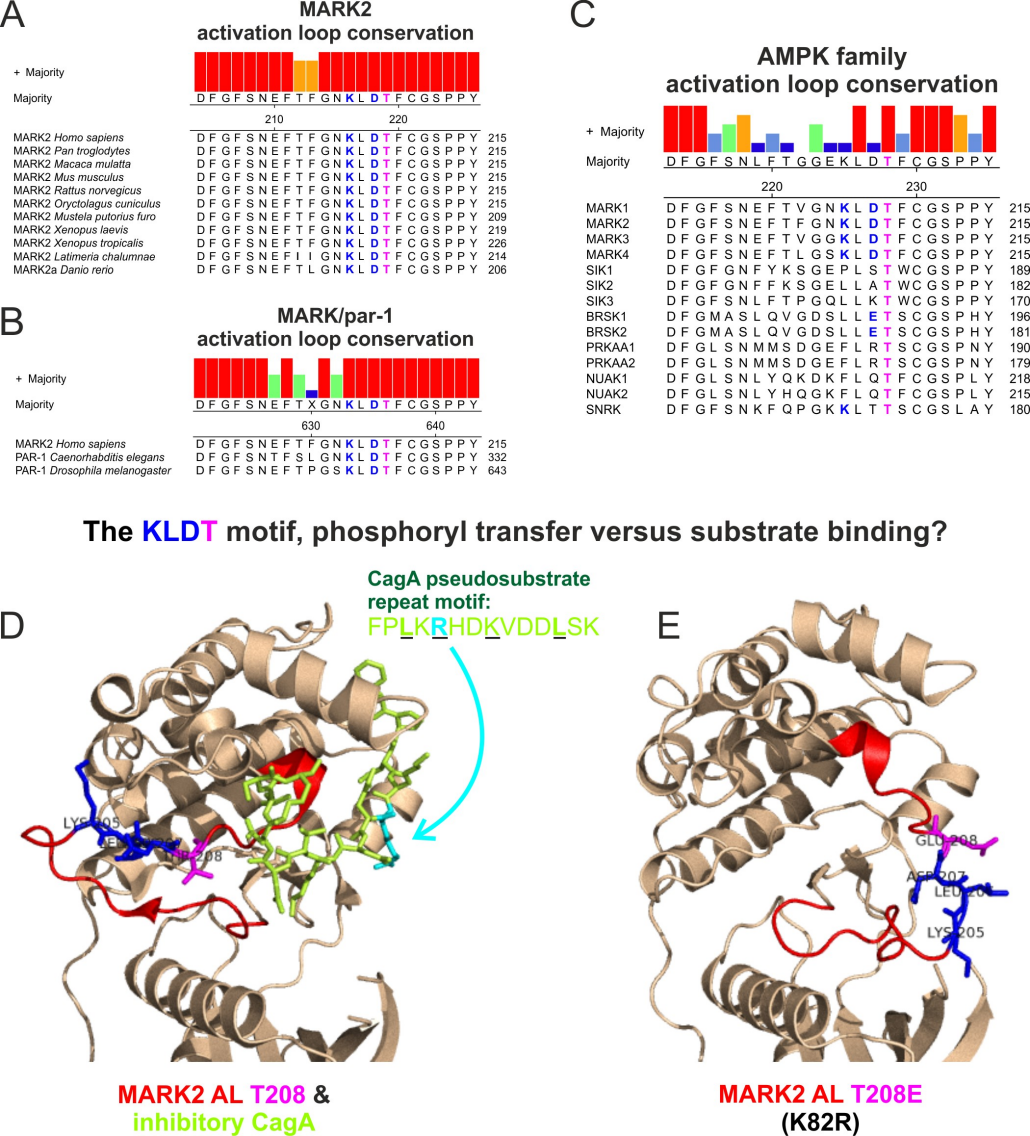
Conservation and putative function of the MARK-specific KLDpT activation loop motif. A) Sequence alignment of the activation loop from different vertebrate MARK2 species. UniProt identifiers (top to bottom): Q7KZI7-1, K7BGW5-1, F6TA28-1, E9QMP6-1, O08679-1, G1TLC6-1, M3YPH0-1, Q8QGV3-1, F6WG24-1, M3XGW1-1, and E7FBX4-1. B) Sequence alignment of *H*. *sapiens* MARK2 activation loop in comparison to *Caenorhabditis elegans* and *Drosophila melanogaster* par-1 kinase. UniProt identifiers (top to bottom): Q7KZI7-1, Q9TW45-1, and Q963E5-1. C) Sequence alignment of the activation loop across *H*. *sapiens* AMPK family members. D) & E) Comparison of two MARK2 protein structures, which indicate conformational flexibility of the activation loop (highlighted in red) and the KLDT motif. Images were generated using PyMOL: D) *H*. *sapiens* MARK2 49–363 in complex with the *Helicobacter pylori* CagA repeat motif (FPL_-5_KR_-3_HDKVDDL_+4_SK; PDB ID 3IEC [43]). E) *Rattus norvegicus* MARK2 49–363 K82R T208E (PDB ID 2WZJ [44]).

AMPK family members have been shown to regulate both canonical AMPK and variant ζXKXGSXXNΨ motifs [2, 30]. We found that the MARK family has an intrinsically higher potency regarding the phosphorylation of ζXKXGSXXNΨ motifs in comparison to SIKs. The ability for MARKs to recognize AMPK and variant motifs appears to be hardwired into their kinase domain, and more specifically, to the KLDpT activation loop motif. Consistent with its importance for MARK activity, the KLDpT motif is conserved among all vertebrate MARKs (Fig 9A) [42]. Indeed, KLDpT is also present in PAR-1 kinases, the invertebrate paralogs of MARKs (Fig 9B). Although the KLDpT motif is unique to the MARK activation loop (Fig 9C), other AMPK family members contain related motifs: LLEpT in BRSK1/2 and KLTpT in SNRK. Although introduction of KLDpT into SIK2 did not alter specificity, future studies should address the extent to which KLDpT-related sequences influence catalytic activity of BRSK1/2 and SNRK against conventional and variant ζXKXGSXXNΨ motifs.

The consensus LXRXXSXXXL motifs inside CRTCs and class IIA HDACs display a greater degree of flexibility than the variant ζXKXGSXXNΨ motifs in tau and MAP2/4. Indeed, these canonical motifs can either naturally lack critical residues at positions -5, -3 or +4 or tolerate mutations at these positions, for example the Ala mutation at -5 Leu of L_-5_NTGGpS273LPDL_+4_ in mCRTC3 had no effect on SIK- or MARK-mediated phosphorylation [10]. A crucial difference between consensus and variant motifs is their biological role, as the AMPK motifs in CRTCs and class IIA HDACs also function in parallel as cellular 14-3-3 binding sites [48], while ζXKXGSXXNΨ phosphorylation alone is sufficient to displace tau and MAP2/4 from microtubules [24, 52]. The 14-3-3 consensus site is described as RSXpSXP [53], and indeed the +2 Pro is found in six out of the seven AMPK motifs in CRTCs and class IIA HDACs. Consistent with their importance for 14-3-3 interaction, while the +2 Pro is critical in L_-5_NTGGpS273LP_+2_DL_+4_, at the site missing the +2 Pro L_-5_NR_-3_TNpS162DSAL_+4_ the -3 Arg was required for phosphorylation and 14-3-3 binding of mCRTC3 [10]. Therefore, the AMPK-like motifs might allow for greater deviations from the consensus as long as they enable efficient 14-3-3 protein binding.

Previous studies documented the influence of activation loop residues on catalytic activity as well as substrate specificity [54], with point mutants having either beneficial or deleterious effects on activity [55, 56]. The KLDpT motif (MARK2 K205L D207A) we identified could modulate activity either via ubiquitous effects on catalytic activity or via modulation of substrate specificity. The KLDpT motif incorporates the phosphorylated activation loop threonine (T208 in MARK2), which is required for cellular activity [3, 50]. Accordingly, changes within the surrounding residues might affect the stoichiometry of threonine phosphorylation and correspondingly the catalysis of the phosphoryl transfer reaction. The phosphorylated KLDpT activation loop sequence may also influence MARKs substrate specificity by enhancing ζXKXGSXXNΨ and LXRXXSXXXL recognition. The structure of substrate-bound MARK has not been elucidated, but MARK2 with unphosphorylated activation loop (T208) has been crystallized in complex with an inhibitory pseudosubstrate (CagA) that resembles a classical AMPK motif (Fig 9D) [43]. Notably, the KLDT208 motif makes no direct contact with CagA in this structure. The structure of another inactive MARK2 (K82R) carrying the activation loop mimic T208E, shows a different outward-facing activation loop conformation in an otherwise rigid kinase domain (Fig 9E) [44]. These results suggest that the MARK2 activation loop retains a high degree of conformational flexibility, which may allow it to participate in substrate recognition. In addition, computational modeling studies of PKA support the notion that catalytic activity and substrate specificity are not mutually exclusive; the phosphorylated Thr inside its activation loop modulates PKA activity in the phosphoryl transfer reaction as well as in the active site conformation [57].

In the course of our ζXKXGSXXNΨ motif studies, we characterized the KA1 domain-mediated interaction of MARK1-3 with CLASP1/2. Indeed, the interaction of MARK3 with CLASP1 and CLASP2 by co-IP has been reported [58] and the MARK2-CLASP2 complex has also been documented in IP-MS experiments [59]. In the former study, CLASP1/2 were found to serve as *in vitro* substrates of MARK3, which phosphorylated these proteins at a conserved and classical AMPK motif (hCLASP1/2: L_-5_QR_-3_SRpS600/370DIDV_+4_) [58]. Although CLASP1 interaction and KA1-dependent membrane localization were dispensable in cells expressing MARK2, these molecular properties may be critical in promoting the localized displacement of tau and MAPs from microtubules. Recent studies with a MARK1”mini” protein consisting of the kinase & UBA domains fused to KA1 revealed that the KA1 domain has auto-inhibitory effects on MARK catalytic activity [60, 61], under conditions when KA1-phospholipid interactions are absent [60]. These results suggest that cell membrane-bound MARK1-3 and microtubule-bound MARK4 are catalytically active, where they mediate the displacement of MAP2/4 and tau from microtubules via ζXKXGSXXNΨ phosphorylation. Following activation of aPKC [15] and other kinases [16], MARKs undergo phosphorylation and 14-3-3 protein binding, which promotes their translocation to the cytoplasm and thereby limits MARK cellular activity. The mechanism by which the phosphorylated MARK-14-3-3 complex interferes with the KA1-mediated phospholipid interaction while at the same time allowing KA1 to bind to and inhibit the N-terminal kinase domain, is unclear. Future studies should reveal signals that modulate cell polarity by controlling MARK subcellular localization and activity.

## Supporting information

S1 Fig Uncropped and unadjusted images for Western blot of Fig 1. A) Corresponds to Fig 1E. B) Corresponds to Fig 1F. Molecular weights were derived from the Precision Plus Protein Dual Color marker (Bio-Rad).
(TIF)

S2 Fig Uncropped and unadjusted images for Western blot of Fig 2. Corresponds to Fig 2D. Molecular weights were derived from the Precision Plus Protein Dual Color marker (Bio-Rad).
(TIF)

S3 Fig Uncropped and unadjusted images for Western blot of Fig 3. A) Corresponds to Fig 3B. B) Corresponds to Fig 3C. Molecular weights were derived from the Precision Plus Protein Dual Color marker (Bio-Rad).
(TIF)

S4 Fig Uncropped and unadjusted images for Western blot of Fig 4. A) Corresponds to Fig 4B. B) Corresponds to Fig 4C. C) Corresponds to Fig 4D. Molecular weights were derived from the Precision Plus Protein Dual Color marker (Bio-Rad).
(TIF)

S5 Fig Uncropped and unadjusted images for Western blot of Fig 5. Corresponds to Fig 5C. Molecular weights were derived from the Precision Plus Protein Dual Color marker (Bio-Rad).
(TIF)

S6 Fig Uncropped and unadjusted images for Western blot of Fig 6. A) Corresponds to Fig 6C. B) Corresponds to Fig 6D. C) Corresponds to Fig 6G. Molecular weights were derived from the Precision Plus Protein Dual Color marker (Bio-Rad).
(TIF)

S7 Fig Uncropped and unadjusted images for Western blot of Fig 7. A) Corresponds to Fig 7C. B) Corresponds to Fig 6E. C) Corresponds to Fig 6F. Molecular weights were derived from the Precision Plus Protein Dual Color marker (Bio-Rad).
(TIF)

S8 Fig Uncropped and unadjusted images for Western blot of Fig 8. A) Corresponds to Fig 8B. B) Corresponds to Fig 8E. Molecular weights were derived from the Precision Plus Protein Dual Color marker (Bio-Rad).
(TIF)
