# Supplementary figures and images for: The KLDpT activation loop motif is critical for MARK kinase activity
Source: PLoS One. 2019 Dec 3;14(12):e0225727. doi: 10.1371/journal.pone.0225727 (PMC6890249; doi:10.1371/journal.pone.0225727)

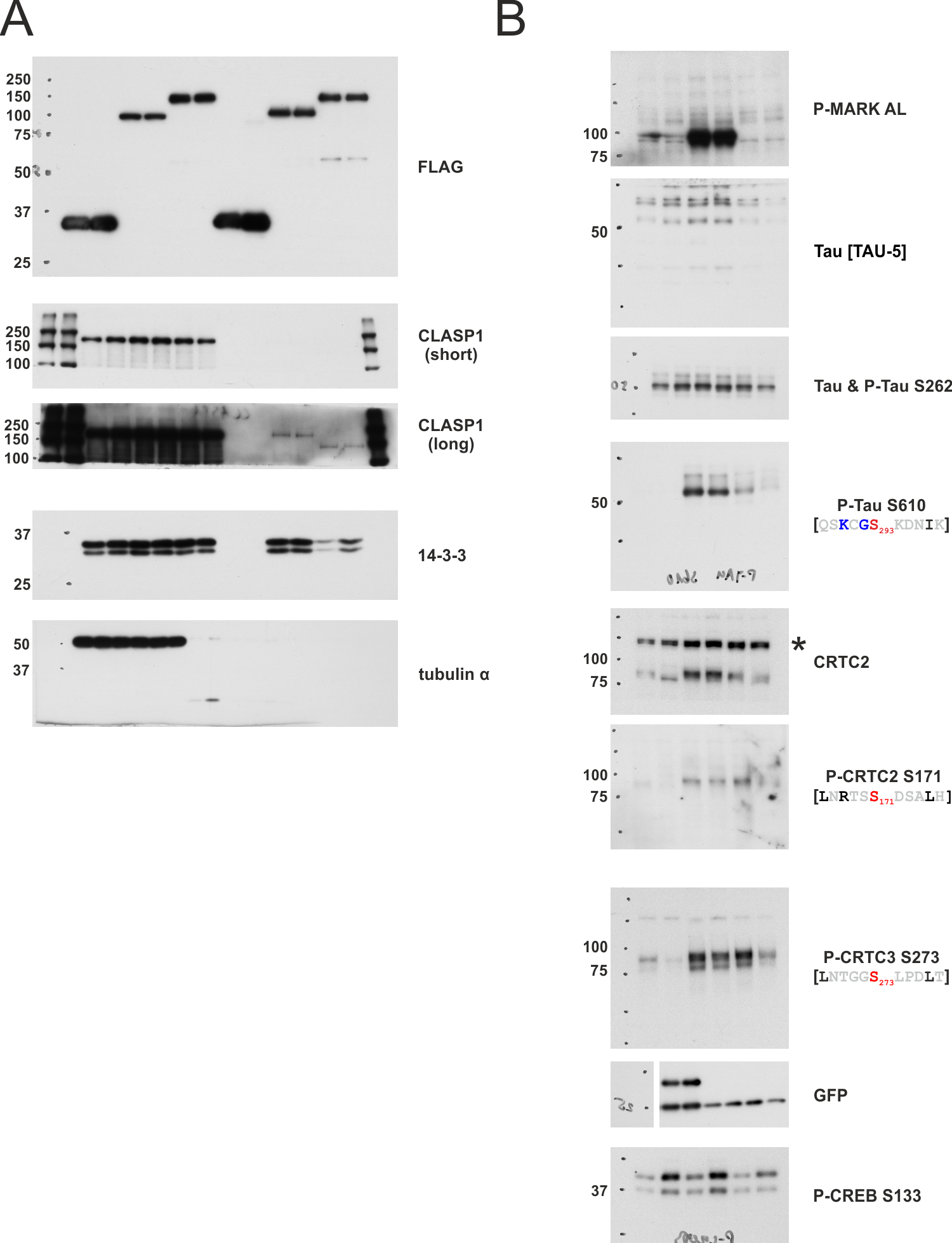

Supplement: S1 Fig — A) Corresponds to Fig 1E. B) Corresponds to Fig 1F. Molecular weights were derived from the Precision Plus Protein Dual Color marker (Bio-Rad). (TIF) [file pone.0225727.s001.tif]

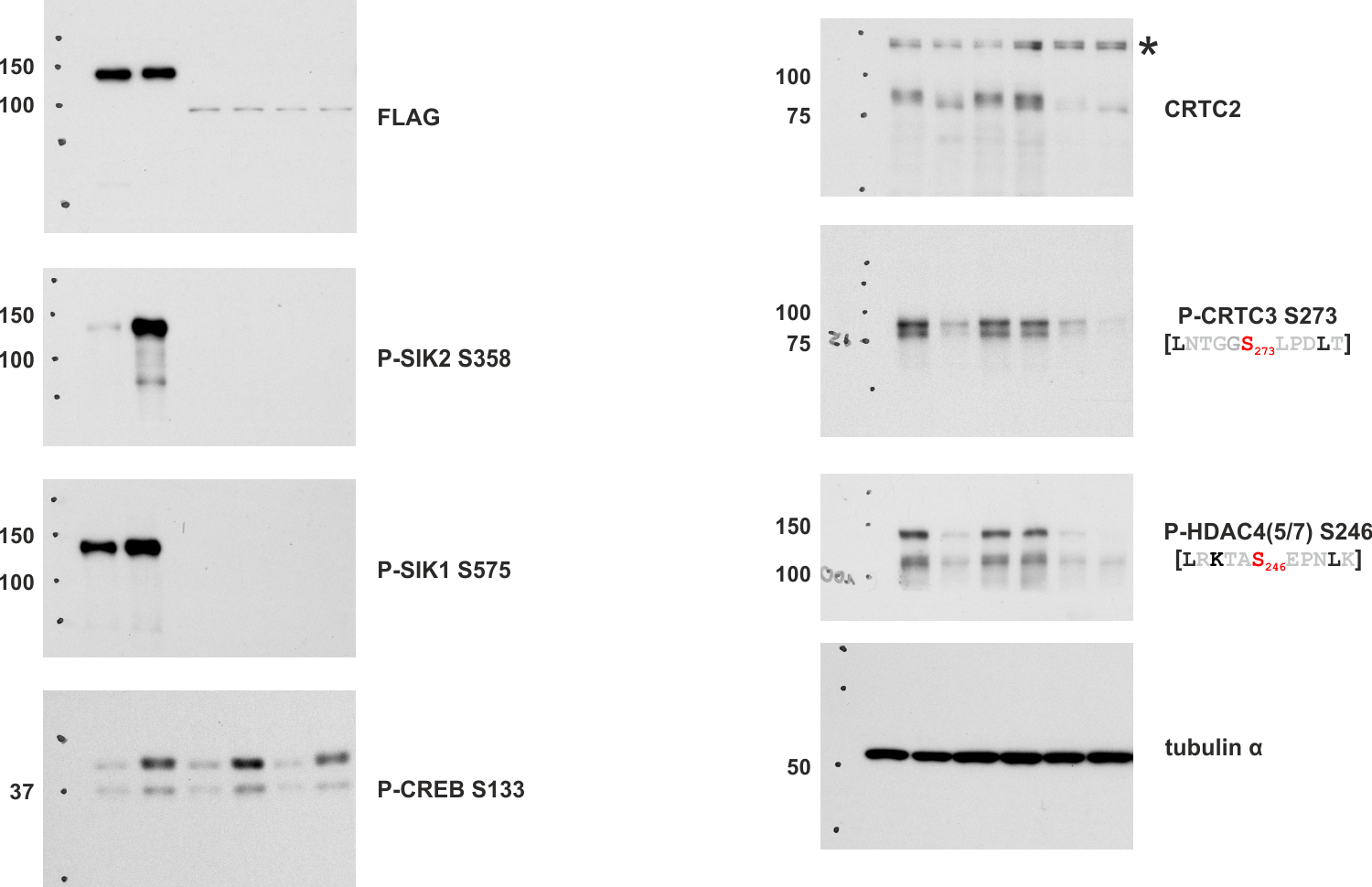

Supplement: S2 Fig — Corresponds to Fig 2D. Molecular weights were derived from the Precision Plus Protein Dual Color marker (Bio-Rad). (TIF) [file pone.0225727.s002.tif]

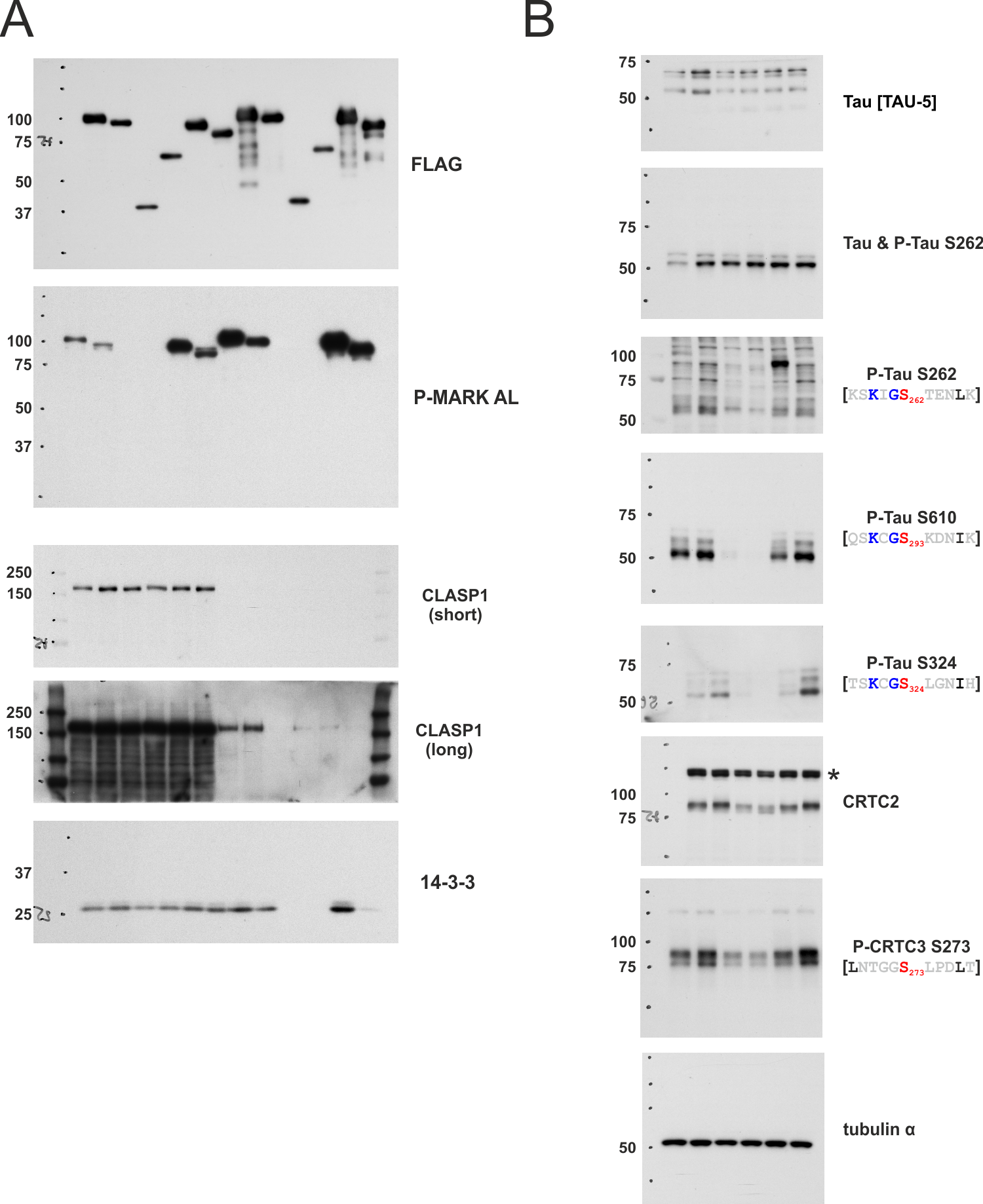

Supplement: S3 Fig — A) Corresponds to Fig 3B. B) Corresponds to Fig 3C. Molecular weights were derived from the Precision Plus Protein Dual Color marker (Bio-Rad). (TIF) [file pone.0225727.s003.tif]

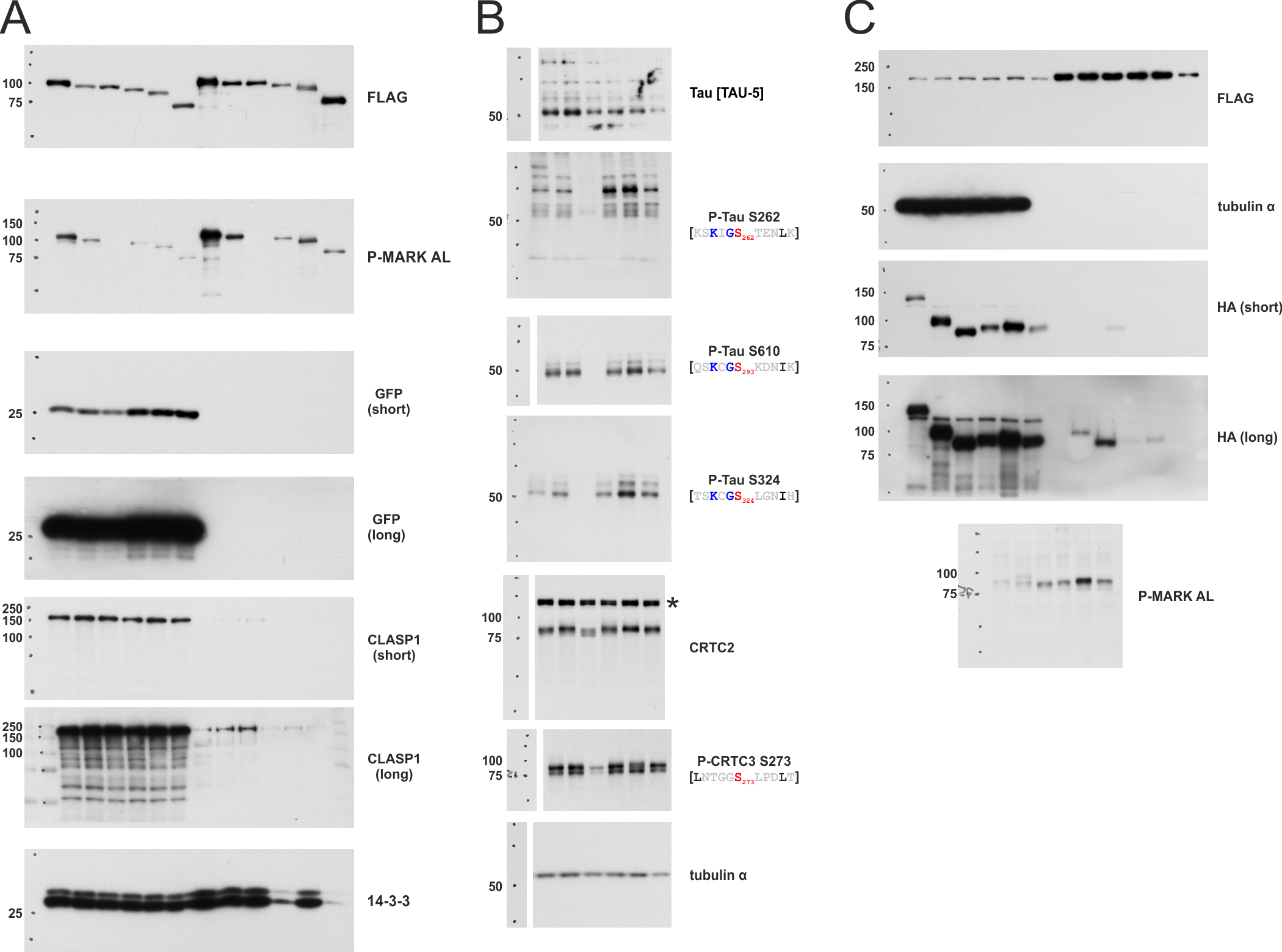

Supplement: S4 Fig — A) Corresponds to Fig 4B. B) Corresponds to Fig 4C. C) Corresponds to Fig 4D. Molecular weights were derived from the Precision Plus Protein Dual Color marker (Bio-Rad). (TIF) [file pone.0225727.s004.tif]

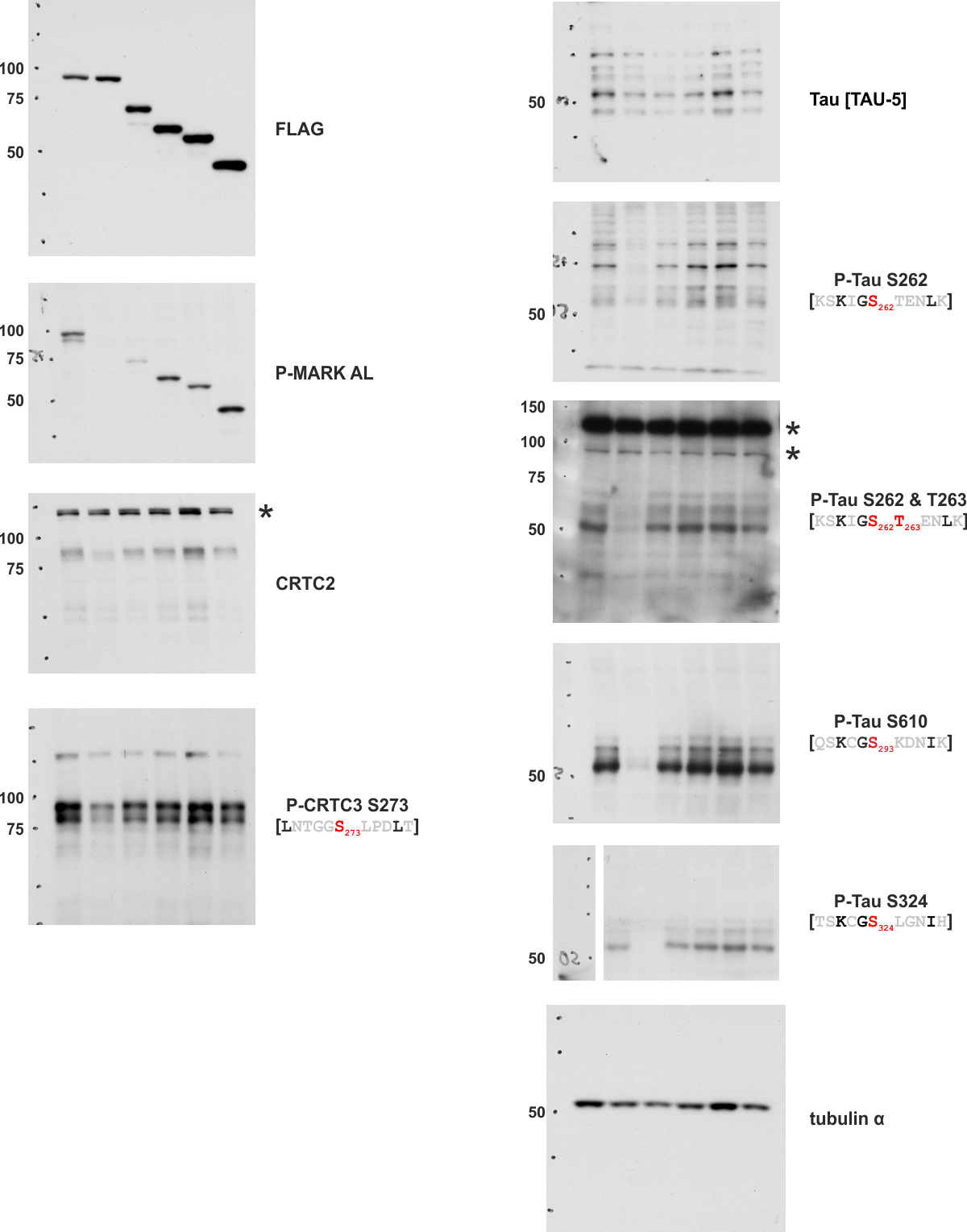

Supplement: S5 Fig — Corresponds to Fig 5C. Molecular weights were derived from the Precision Plus Protein Dual Color marker (Bio-Rad). (TIF) [file pone.0225727.s005.tif]

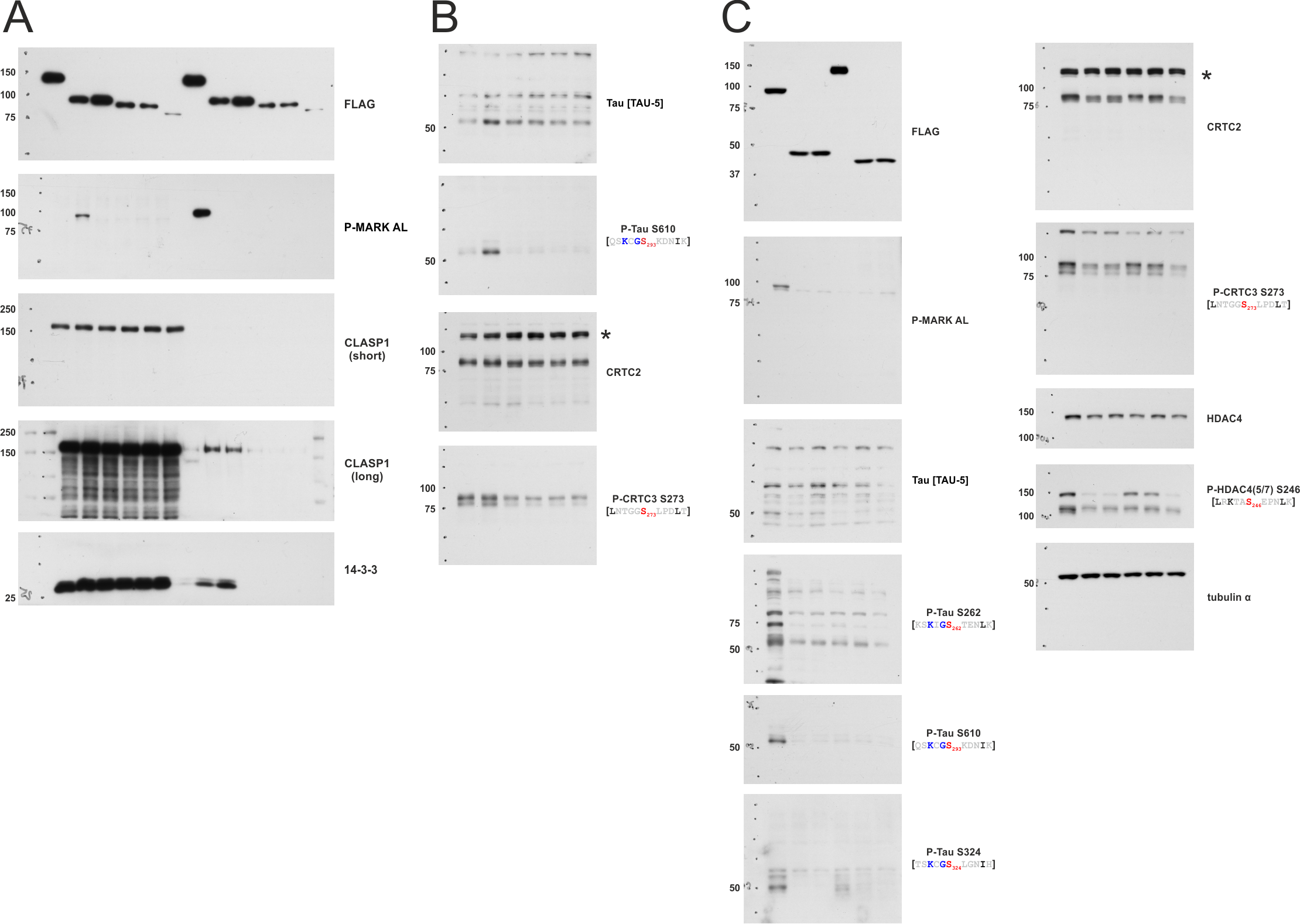

Supplement: S6 Fig — A) Corresponds to Fig 6C. B) Corresponds to Fig 6D. C) Corresponds to Fig 6G. Molecular weights were derived from the Precision Plus Protein Dual Color marker (Bio-Rad). (TIF) [file pone.0225727.s006.tif]

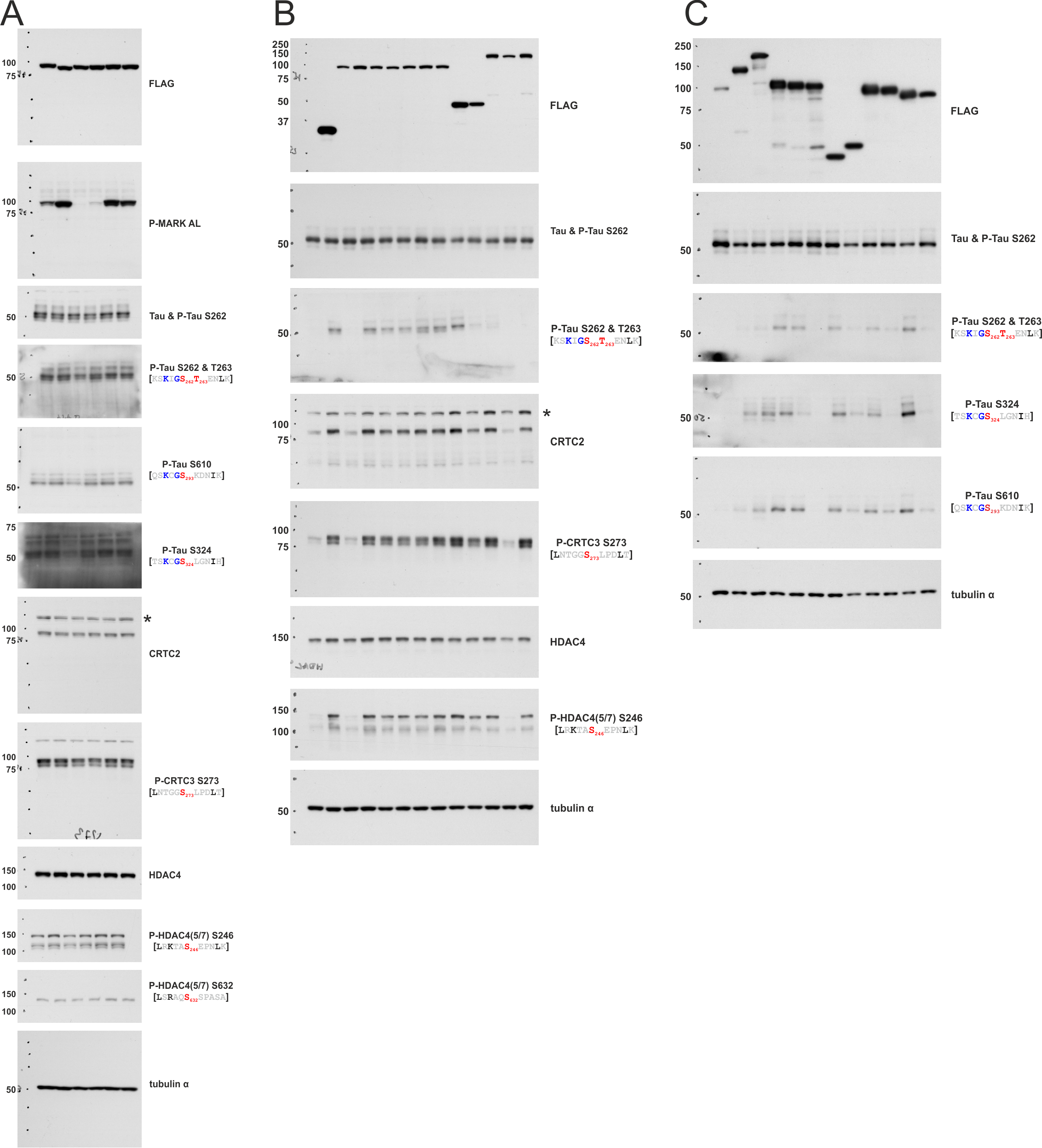

Supplement: S7 Fig — A) Corresponds to Fig 7C. B) Corresponds to Fig 6E. C) Corresponds to Fig 6F. Molecular weights were derived from the Precision Plus Protein Dual Color marker (Bio-Rad). (TIF) [file pone.0225727.s007.tif]

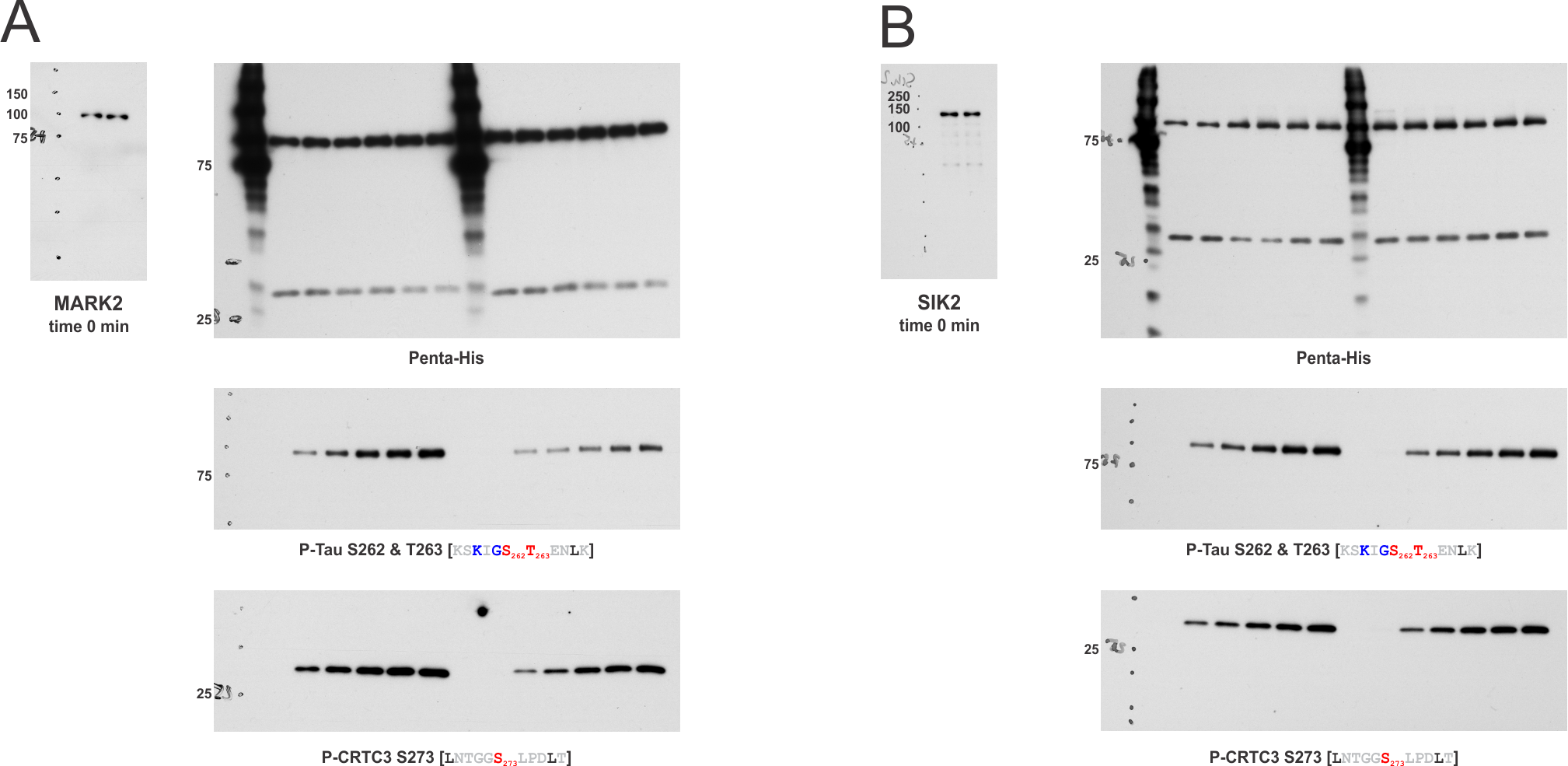

Supplement: S8 Fig — A) Corresponds to Fig 8B. B) Corresponds to Fig 8E. Molecular weights were derived from the Precision Plus Protein Dual Color marker (Bio-Rad). (TIF) [file pone.0225727.s008.tif]
